# Predicting cognitive decline in a low-dimensional representation of brain morphology

**DOI:** 10.1038/s41598-023-43063-4

**Published:** 2023-10-05

**Authors:** Rémi Lamontagne-Caron, Patrick Desrosiers, Olivier Potvin, Nicolas Doyon, Simon Duchesne

**Affiliations:** 1 Département de médecine, Université Laval, Quebec, QC G1V 0A6 Canada; 2 Centre de recherche CERVO, Quebec, QC G1J 2G3 Canada; 3 Centre interdisciplinaire en modélisation mathématique, Université Laval, Quebec, QC G1V 0A6 Canada; 4 Département de physique, de génie physique et d’optique, Université Laval, Quebec, QC G1V 0A6 Canada; 5 Département de mathématiques et de statistique, Université Laval, Quebec, QC G1V 0A6 Canada; 6 Département de radiologie et médecine nucléaire, Université Laval, Quebec, QC G1V 0A6 Canada

**Keywords:** Neurodegeneration, Computational neuroscience, Neural ageing, Alzheimer's disease, Scientific data

## Abstract

Identifying early signs of neurodegeneration due to Alzheimer’s disease (AD) is a necessary first step towards preventing cognitive decline. Individual cortical thickness measures, available after processing anatomical magnetic resonance imaging (MRI), are sensitive markers of neurodegeneration. However, normal aging cortical decline and high inter-individual variability complicate the comparison and statistical determination of the impact of AD-related neurodegeneration on trajectories. In this paper, we computed trajectories in a 2D representation of a 62-dimensional manifold of individual cortical thickness measures. To compute this representation, we used a novel, nonlinear dimension reduction algorithm called Uniform Manifold Approximation and Projection (UMAP). We trained two embeddings, one on cortical thickness measurements of 6237 cognitively healthy participants aged 18–100 years old and the other on 233 mild cognitively impaired (MCI) and AD participants from the longitudinal database, the Alzheimer’s Disease Neuroimaging Initiative database (ADNI). Each participant had multiple visits ($n \ge 2$), one year apart. The first embedding’s principal axis was shown to be positively associated ($r = 0.65$) with participants’ age. Data from ADNI is projected into these 2D spaces. After clustering the data, average trajectories between clusters were shown to be significantly different between MCI and AD subjects. Moreover, some clusters and trajectories between clusters were more prone to host AD subjects. This study was able to differentiate AD and MCI subjects based on their trajectory in a 2D space with an AUC of 0.80 with 10-fold cross-validation.

## Introduction

A proper understanding of brain morphology trajectory during cognitively healthy aging can be leveraged to detect departures due to neurodegeneration (e.g. from Alzheimer’s disease (AD)) and therefore serve as an early indicator of incipient dementia. As the socio-economic costs of AD increase with the aging of global populations^1,2^, early detection becomes the only approach that allows for the possibility of interventions able to reduce the risk of conversion to dementia.

Brain morphology can be assessed via magnetic resonance imaging (MRI)^3–6^. Morphometry has been shown to be a sensitive marker of neurodegeneration^7,8^. However, normal aging cortical decline and high inter-individual variability complicate the comparison and statistical determination of the impact of AD-related neurodegeneration on trajectories. Further, the high-dimensional nature of morphometry (whereas the available measurements routinely surpass the number of study participants) necessitates the use of a transformation into lower-dimensional spaces in order to perform statistical analyses, modeling or machine learning^9–12^. In these resulting spaces of low dimensionality, the task then turns to the identification of criteria with which to determine whether a given brain morphology trajectory is pathological or not, which implies having comparative instances of both successful and pathological natures.

High-to-low dimensionality transformations have been carried out via a number of approaches in the past. In some instances, dimensionality reduction or machine learning algorithms were applied directly to the raw voxel data^13–17^. Other approaches first use tools (e.g. FreeSurfer^18^) to extract features such as volumes, thicknesses or surface areas for individual brain structure, then perform dimensionality reduction on the feature vectors^19–23^. While the first family of approaches may yield classifiers with adequate accuracy, the lower-dimensional space and resulting classification functions are usually difficult to interpret. On the other hand, the second family of approaches allows the association between principal dimensions to interpretable features with biological meaning^24^, which in turn might suggest intervention strategies. In recent years, the brain age field of research has used both approaches to predict biological age for the whole spectrum of cognitive health with successful results. In turn, this technique has also shown potential for the prediction of cognitive decline^25–29^.

Uniform Manifold Approximation and Projection (UMAP) is a novel entry in the domain of high-to-low dimensionality reduction techniques. First published in 2018^30^, UMAP’s backbone rests on Riemannian manifold theory and topological data analysis. In a high-dimensional space in which each brain is a point, a manifold is a lower dimensional embedded space. UMAP is a method for projecting brains (points in the full space) onto a 2-D or 3-D manifold in a way that preserves essential relations but makes visualization possible. Being a Riemannian manifold, the space in which the brain points live has a distance function (Riemannian metric) that is non-Euclidean, and UMAP can project them onto visible 2 or 3D manifold in a way that preserves their distance relations under that metric. To accomplish this, UMAP uses two hyperparameters: n_neighbors and min_dist. The former describes how global the preserved relationships are, while the latter defines the distances between points in 2-D. Furthermore, its main advantage over other nonlinear methods, like t-distributed Stochastic Neighbor Embedding (t-SNE)^31^ or nonlinear principal component analysis^32^, comes from its agility, performance and conservation of global structures. Arising from Riemannian mathematics, the algorithm is highly generalizable and doesn’t necessitate strict priors on the data, such as the features being linearly dependent between each other. As to not imbue priors on the low-dimension embedding, UMAP is a good candidate. Further, as opposed to highly nonlinear deep learning networks, a UMAP based method is interpretable through visualization. It is therefore a suitable technique to handle the nonlinear nature of brain morphological trajectories and embed our data vectors in a lower-dimensional space. Examples of similar applications can be found in Campbell et al. and Becht et al.^33,34^

In this article, we present our work on brain morphology trajectory estimation on a number of well-known, publicly available datasets composed of individuals across the spectrum of cognitive health. Our general goal is to use UMAP to generate a reference low-dimensional representation of morphology from vectors of features describing the thicknesses of different brain regions; then project in this space data from individuals across the spectrum of cognitive decline. A discretization scheme alongside probabilistic analysis of trajectories is used to achieve the best possible accuracy at predicting the longitudinal cognitive outcome in individuals. As a first experiment, we built the reference space using a dataset composed of cognitively healthy (CH) individuals, in which we projected data on subjects with mild cognitive impairment (MCI; a prodromal state of AD) that either progressed to dementia due to probable Alzheimer’s disease (MCI-AD) or remained cognitively stable (MCI-MCI). In a second experiment, we used data on MCI-AD and MCI-MCI individuals to form the reference space as a basis for classification. In both experiments we tested the association with aging.

## Methods

**Figure 1:**
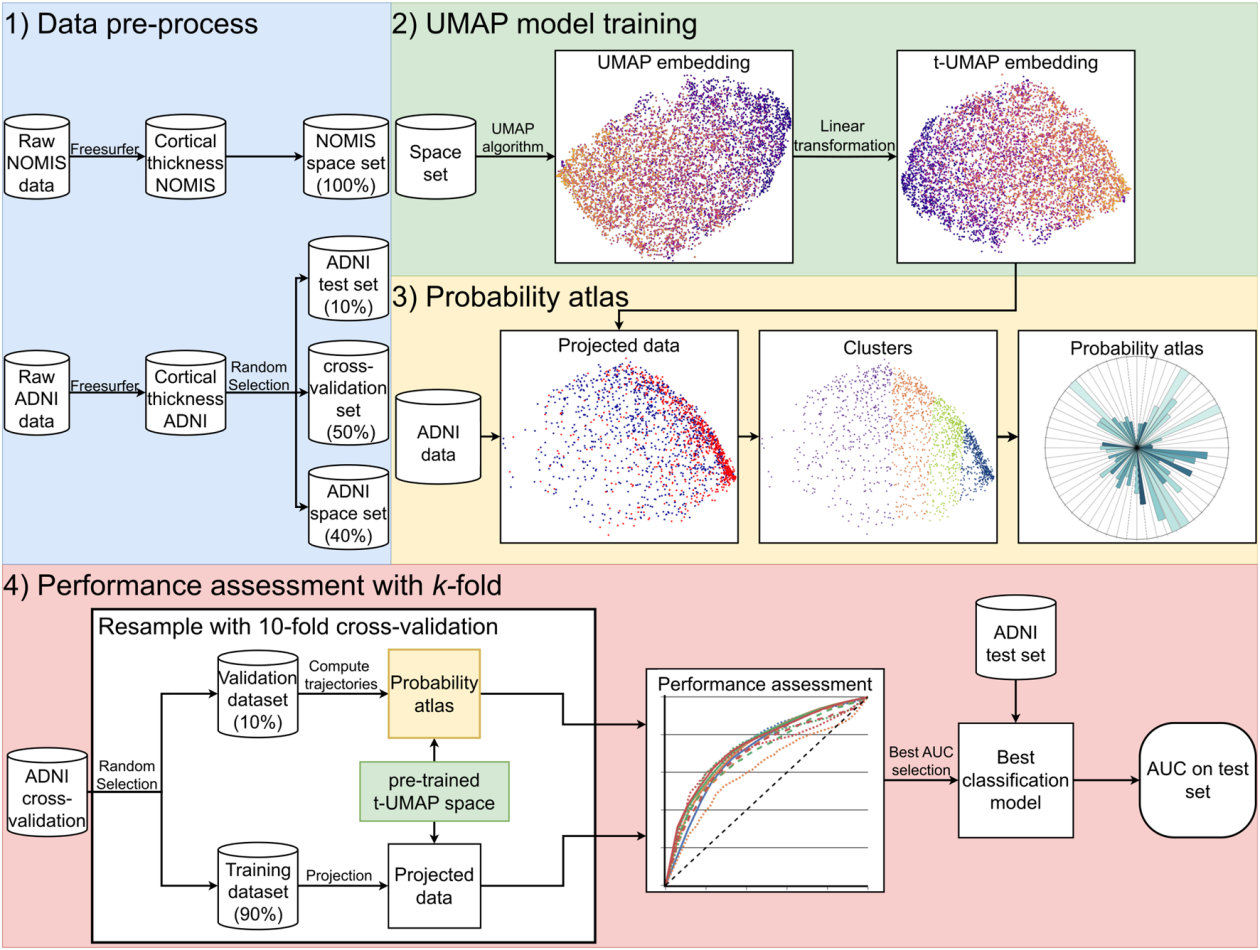
Methodological pipeline for the prediction of cognitive outcome. (1) We separate the different datasets (NOMIS and ADNI) into the necessary subsets (test, cross-validation and space set). (2) Different UMAP models are trained on one of the two space sets which results in the an embedding referred to as “UMAP embedding”. This embedding is linearly transformed so its axes are align with its principal axes. This transformation in turns results in a new embedding referred to as “t-UMAP embedding”. The latter embedding is fed to the probability atlas algorithm. The figures displayed under “UMAP embedding” and “t-UMAP embedding” were made using the NOMIS dataset with n_neighbors = 20 as the UMAP parameter. (3) The probability atlas algorithm reduces the longitudinal data (ADNI for this paper) using the previously trained embedding, clusters the resulting data and computes the population and probability associated with each trajectory. (4) Finally, the performance is assessed through 10-fold cross-validation using the “cross-validation set”, and the model with the best AUC is tested on the test set to generate the final accuracy of the method.

Figure 1 shows a diagram of the data processing pipeline used for this research, described in more detail in the following section.

### Ethics

Approval for the study was obtained from the Ethics review committee of the CERVO Brain Research Center (NSM-2021-2022). Approval for this study was obtained from the local ethics board and informed consent for all participants were obtained for all datasets in this study (see Acknowledgements). All methods were carried out in accordance with relevant guidelines and regulations.

### Participants

#### NOMIS

The NOMIS database^35^ was used to train UMAP’s dimensionality reduction model. The database is composed of a total of 6237 CH participants (3,556 women), aged 18–100 years old (median 56 y.o.), and aggregated from 26 different databases^35^. Participants had no history of: psychotic disorders, mood, anxiety, post-traumatic stress, or substance abuse/dependence disorders; neurodegenerative and neurological disorders; head injury with loss of consciousness/amnesia; lead poisoning; or geriatric depression. Since those participants were scanned once, the NOMIS data is cross-sectional.

#### ADNI

The Alzheimer’s Disease Neuroimaging Initiative (ADNI) database (ADNI1, GO, 2 and 3)^36^ was used to test the UMAP model for the prediction of cognitive decline. Launched in 2003 as a public-private partnership, led by Principal Investigator Michael W. Weiner, MD, ADNI’s goal has been to test whether serial MRI, positron emission tomography, other biological markers, and clinical and neuropsychological assessment can be combined to measure the progression of MCI and early AD. For up-to-date information, see www.adni-info.org. All ADNI participants with longitudinal time points and at least one follow-up after the baseline scan were selected for this project, for a total of 1354 participants (604 women). We used ADNI’s clinical diagnostic categorization at baseline (526 CH; 828 MCI; 0 AD) and last available follow-up (477 CH, 537 MCI; 340 AD), see Supplementary Table S5 for more details.

#### Diagnostic criteria

For the first ADNI cohort, the criteria for classification between CH, MCI and AD were based on memory loss, the Mini-Mental State Examination (MMSE), the Clinical Dementia Rating score and one paragraph from the Logical Memory II subscale of the Wechsler Memory Scale-Revised. For memory loss, participants needed to have complained about lapses in their memory to be classified as MCI or AD, while CH subjects did not experience memory lapses. With the MMSE, CH and MCI subjects had to have a score between 24 and 30, and for AD, 20–26. Cognitively healthy participants had to have a Clinical Dementia Rating score of 0, MCI 0.5 with the memory box score being 0.5 and AD a score of 0.5 or 1. For the last test, result depended on education. CH participants had a score $\ge 9$ for 16 years of education, $\ge 5$ for 8–15 years of education and $\ge 3$ for 0–7 years of education. In the other hand, MCI and AD subjects had to have a score $\le 8$, $\le 4$ and $\le 2$ for the same education groups^37^.

#### Outcome

Throughout this article, we will refer to the evolution of clinical diagnostic categories as trajectory types. For instance, a subject with a MCI diagnostic at their first and last MRI was considered “stable” (MCI-MCI) while another who developed AD at a later stage would be a “converter” (MCI-AD).

### MRI data

#### NOMIS

Each NOMIS participant had an available T1-weighted MRI. Because the origin of the data is multiple, the protocol, MRI manufacturer and magnetic field strengths used were not uniform throughout the sample. Regarding manufacturers, 1160 scans (18.60%) were made with a Philips Medical Systems MRI (Best, Netherlands), 3853 (61.77%) with a Siemens Healthcare (Erlangen, Germany) scanner and 1224 (19.63%) using GE Healthcare’s products (Milwaukee, WI). Of these scans, 4778 were done at magnetic field strengths of 3.0 Tesla and 1459 at a field of 1.5 Tesla.

Part of the data used in the preparation of this article were obtained from the Parkinson’s Progression Markers Initiative (PPMI) database. For up-to-date information on the study, visit www.ppmi-info.org.

#### ADNI

Participants were scanned on different MRI units albeit with a standardized protocol^38^. In total, there were 7414 scans and 4157 of those from MCI individuals at baseline. From those, the distribution of scans was similar to NOMIS with 547 (13.16%) using Philips Healthcare products, 2048 (49.26%) on Siemens Medical Systems units and 1562 (37.58%) on GE Healthcare. MRIs were taken with two field strengths; 2157 at 3.0 Tesla and 2000 at 1.5 Tesla. The age distribution was similar between MCI-AD (range: 55.1–95.9 y/o; average 76.1, SD 7.2 y/o) and MCI-MCI indviduals (range: 55.2–97.4 y/o; average 74.6, SD 7.7 y/o). For a full list of ADNI’s subjects, refer to Supplementary Tables S6–S10.

#### ADNI pre-processing

ADNI being a longitudinal, multi-centric study, some subjects were scanned every six months while others every year, with different lengths of follow-up (median of 10.2 months, mean of 11.04 months and a standard deviation of 7.8 months). Thus, before being processed, ADNI longitudinal data was binned into standardized time step of 1 year (+/− 5 months), based on the distribution of time steps in the index. This process and its impact on the data is documented in Supplementary Figs. S1–S4. This served to make trajectories comparable among participants. Further, in ADNI1/GO, some participants were scanned initially at a field of 1.5 T, but at every other follow-up at 3.0 T. In these cases the first scan was discarded in order to not bias morphometry estimates by scanner and field strength changes. In fine, the ADNI sample consisted of 1780 scans from 549 MCI subjects with at least one follow-up, an average age of 74.6 (7.8) y/o for the MCI-MCI and 76.8 (7.3) y/o for MCI-AD. The distribution of regularized time steps had a median of 1.01, a mean of 1.00 and a standard deviation of 0.14.

#### Image processing

Each T1-weighted MRI was processed using *FreeSurfer* version 6.0, a free open-source software used to quantify brain anatomy. *FreeSurfer* encompasses multiple brain atlases to produce brain segmentation. We selected the Desikan-Killiany-Tourville atlas^39^ (DKT, aparc.DKT.stats file) for the cortical segmentation. The MRI data were processed using the “recon-all -all” command in *FreeSurfer* with the fully automated directive parameters (no manual intervention or expert flag options) on the CBRAIN^40^ platform to extract thicknesses for all defined atlas regions.

The processing techniques used by FreeSurfer^18^ to generate the cortical models consisted mainly of motion correction, removal of non-brain tissues using watershed/surface deformation procedure, automated Talairach transformation, intensity normalization, tessellation of the gray matter white matter boundary, automated topology correction, and surface deformation following intensity gradients to accurately place the tissue boundaries between gray/white matter and gray/cerebrospinal fluid. Additional processes are available following the cortical model : surface inflation, registration to a spherical atlas and parcellation of the cerebral cortex into units. These allow for a better representation of cortical thickness by using the intensity values and the whole information from the volume segmentation. Thickness is calculated using the closest distance from the gray/white boundary to the gray/CSF boundary at each vertex on the tessellated surface^35^.

For this project, we average regional cortical thickness per region as a proxy of cortical neuronal degeneration, as validated in the work of Cardinal and Belathur^41,42^. This average was turned into a Z-score using the NOMIS tool, which normalizes the data based on a two-step regression model taking into account image quality and head size (eTIV), and sex. Since age is an important factor in AD it was not used here to normalize data. Hence, for each participant, expected measure values were generated in the form of Z-scores that demonstrate deviations from the normative NOMIS values.

### Data analysis

#### Low dimensional reduction

According to the DKT atlas, the cortex was divided in 31 regions for each of the left and right hemispheres, and hence each data point was represented by a 62-dimensional vector. To facilitate predictive modeling, dimensional reduction of the data with UMAP was an important step. UMAP is a nonlinear algorithm based on Riemannian geometry and algebraic topology used for dimension reduction of high-dimensional datasets. To accomplish this, UMAP first computes the topology of the manifold as a *k*-nearest neighbor graph. The graph is weighted by the probability that two points are connected based on their distance computed with the Riemannian metric. The further two points are from each other, the least probable they are to be connected. For a given node, the number of nearest neighbors with which distances can be calculated are determined by the hyperparameter n_neighbors, denoted by *k* in the mathematical and computer science literature^30,31^. The other hyperparameter, min_dist, describes the minimum distance between points in the reduce space. When, UMAP tries to project the data from a high Riemannian space onto a lower Euclidean space, it does so by respecting the minimal possible distance between points in that space. Finally, the topological graph is generated for the low-dimension space, and by optimizing the cross-entropy between the lower and higher dimension topology, the algorithm finds the projection that represents the most the data topology^43^(further details are included in McInnes^30^). As previously mentioned, the two hyperparameters give the algorithm generalizability while allowing us to find the embedding that fits our need. In our first experiment, we generated a low-dimensional manifold using UMAP by reducing the 62-dimensional cortical thickness data from NOMIS to *m*-dimensions (with its axes being UMAP0, UMAP1, ..., UMAP$m-1$), where $m\ll 62$, using $\texttt {min\_dist}=0$ and different n_neighbors values of 2–100 (Figs. 3 and 4). We initially verified if $m=2$ or $m=3$ was the better target dimension for our problem. To do so, the explained variance of each dimension was calculated to verify if the third dimension was useful. We then used the trained model to transform the entire ADNI data in the new *m*-D space.

In our second experiment, we created a low dimension embedding model using the ADNI database only. For this model, we randomly selected 40% of the subjects in the ADNI dataset to generate the UMAP embedding, in which we projected data from the remaining participants, performed clustering, and calculated the probability of AD associated with trajectories. For both models, no feature selection was made on the data so that UMAP has full information to maximize the distance between MCI-MCI and MCI-AD and see if relevant cortical regions can be extracted.

We hypothesized that the first model would encompass the characteristics of normal aging which could distinguish AD and MCI patients, but the second model could directly represent the morphological decline related to dementia and thus be more predictive of the cognitive outcome. In the rest of the paper, we will use *NOMIS embedding* to refer to the embedding trained on NOMIS and *ADNI embedding* to refer to UMAP models trained on ADNI.

#### Transformation for axis analysis

In this application of the nonlinear UMAP method, it is difficult to interpret axes in terms of the features (thickness of cortical regions). Remark that algorithms such as PCA provide a linear combination of features to generate axes, while with UMAP these relations come from correlations. Moreover, the UMAP embedding doesn’t impose an orientation on the data and thus, any rotation is equally valid. Therefore, after having applied the UMAP algorithm, we used a linear transformation over the UMAP embedding to rotate the data such that its principal axes could explain most of the variance in the embedding. As described by the principal component analysis, this rotation is given by the singular value decomposition of the embedding^44^. This method allowed us to determine which features correlated the most with UMAP embedding axes. These transformed axes are referred to as t-UMAP. Because aging is a strong confounder in the study of dementia, we tested whether the algorithm implicitly embedded age in both NOMIS and ADNI embedding representations. To test this, we used Pearson’s correlation between age and t-UMAP vectors. We further tested if there were correlations between the axes and the thickness of the different brain regions.

#### Clustering

In either embedding experiment, the low-dimensional t-UMAP space was then parcellated into data clusters, each containing the same number of data points, in order to achieve similar statistical power in our trajectory analyses. The clustering was done on the t-UMAP coordinates only. To this end, we adapted *scikit-learn*’s *k*-means algorithm^45^ to our need of segmenting the data in equal groups.

A number of clusters (*C*) can be specified by the user and the number of data points in each cluster (*n*) is determined from the overall size of the data set. The clusters’ centroids were initialized using *scikit-learn*’s *k*-means++ algorithm^46^. A distance matrix between each pair of points and centroids was then computed, and a probability matrix calculated. The entries of the latter matrix describe the probability that a given data point (row) belongs to a given cluster (column). These probabilities are based on the distances between data points and the centroids according to the formula: $\textbf{W}_{ij} = 1 - \textbf{D}_{ij} / \textbf{V}_i$ where $\textbf{W}$ is the probability matrix, $\textbf{D}$ is the distance matrix and $\textbf{V}$ is a column vector where each element is the maximum value of the corresponding row in $\textbf{D}$. The matrix entry has a value of 0 if the corresponding centroid is the furthest from the data point.

For each cluster, the *n* likeliest points were then assigned to it. If the number of data points was not divisible by *C*, the unassigned data points were assigned to their likeliest cluster.

An iterative process followed, in which, at each iteration, centroids’ position and their distances from every point were calculated; probabilities for a point to switch cluster were ordered; and assignments performed. The process was run until the inertia (within-cluster sum-of-squares of the distances between points and their closest centroid) could not be minimized further, or a maximum number of iterations was reached^45^.

### Probabilistic Trajectory

**Figure 2:**
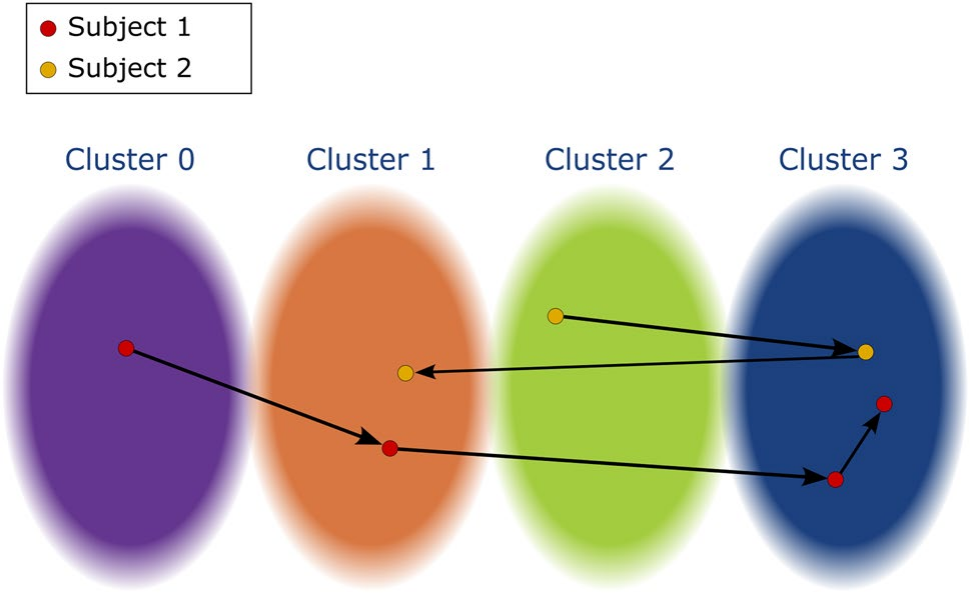
Defining trajectories. An arbitrary embedding with four clusters. The arrows represent the direction in time for each subject. Given an embedding with clusters, we can define the longitudinal trajectory of subjects as follows. In this example, subject 1 would have a full trajectory of [0-1-3-3] while subject 2 [2-3-1]. The trajectory of length 1 would be [3] for subject 1 and [1] for subject 2 and the trajectory of length 3 would be [1-3-3] for subject 1 and [2-3-1] again for subject 2. Subject 2 wouldn’t have a trajectory of length 4 and subject 1’s would be equivalent to its full trajectory.

In the *m*-dimensional space computed from the embedding data, we projected the remaining ADNI data points and segmented them into identical-size clusters, after which we turned our attention to the problem of trajectory prediction.

We first defined as a trajectory the ordered sequence of clusters through which a participant’s data belonged in its longitudinal course. Figure 2 shows a simplified representation of an embedding with four clusters. As shown in the figure, we defined a subject’s trajectories from their latest data point, meaning a trajectory of length 3 is composed of the 3 oldest datum for a subject. To characterize trajectories at the group level, in every possible trajectory, we computed the probability that an individual in this trajectory developed AD. First, given an embedding with *C* clusters, the total number of trajectories *T*, for every possible trajectory of length one to *L*, is given by $T = \sum _{l=1}^{L}C^l$. Each set of *T* trajectories is initialized with a null probability. The probabilistic atlas was then generated, in which every entry was composed of a trajectory and its associated AD progression probability. Then, for every participant, we pieced together cluster trajectories for the last, two last and three last data points, thus creating trajectories of length one, two and three steps for the participants. Depending on the final diagnosis at the last step (i.e. whether the participant remained stable or progressed to AD), the probability of this trajectory with respect to AD was updated consequently. Once the whole dataset was processed, we had an atlas tying the probability of developing AD to each possible trajectory of lengths 1–3.

A disadvantage of this approach is that empty trajectories (paths not taken by any participant) were evaluated as paths with a probability of zero, even though no data were available. These trajectories cannot be tossed aside since future participants might follow such trajectories, and a statistic will be required to “predict” diagnosis. We then arbitrarily chose for these trajectories to remain at a value of zero.

Given that ADNI has 949 MCI individuals at baseline, $C^l$ must be $\ll 949$ so that trajectories of length *l* are reasonably populated. Because of this limitation, we empirically limited the length of a trajectory to the last three data points (i.e., three years before diagnostic of AD) and the maximum number of clusters to 7, which gave us a maximum of 343 unique trajectories. Since the subjects are not uniformly distributed between every trajectory, as shown in Supplementary Figs. S5 and S6, we could afford $C^l$ to be $\approx 1/3$ of the number of subjects. Indeed a lot of trajectories will never be used and can thus be discarded. Supplementary Fig. S5 shows that $C=7$ and $l=3$ gives 108 used trajectories. Given larger datasets, these limitations could be lifted.

### Statistical analysis

We performed a sensitivity analysis using 10-fold stratified cross-validation as follows. First, we separate the ADNI dataset into three groups : the test set (10% of total MCI-MCI and 10% of total MCI-AD subjects), the space set (40% of total MCI-MCI and 40% of total MCI-AD subjects) and the cross-validation set. The latter is then separated using 10-fold stratified cross-validation.

For the NOMIS embedding, only the test and cross-validation set were used. These groups were projected in the transformed UMAP space, created by the NOMIS dataset. One of the fold was chosen as the validation set and the rest were used as the training set. The cluster segmentation algorithm was then applied on the training group, and the probabilistic atlas is calculated as defined above. We then extracted the final results using the validation group. For each participant, we calculated the probability of progressing to AD given their last 1, 2 and 3 data points. We then set an arbitrary threshold to binarize predictions (e.g., every prediction with a probability higher than a threshold of 0.5 would be qualified as an AD subject). We computed a receiver operating characteristic (ROC) curve for each trajectory length (1, 2, or 3) by varying the value of this threshold from 0 to 1, calculating the true positive rate (*sensitivity*), false positive rate ($1- specificity$) and area under the curve (AUC). The process is done on different values of n_clusters. The best model was chosen from this and was then applied to the test set to calculate the actual accuracy of the model.

As for the model generated from ADNI embedding, we used the exact same test, space, and cross-validation sets. In this case, the space set was used to generate the ADNI embedding. Following this embedding, the test and cross-validation set were reduced and the latter separated using 10-fold stratified method. From that point, the same cross-validation process was applied. Finally, the test set was clustered using the trained model and a sensitivity analysis performed.

## Results

### UMAP transformation

**Figure 3:**
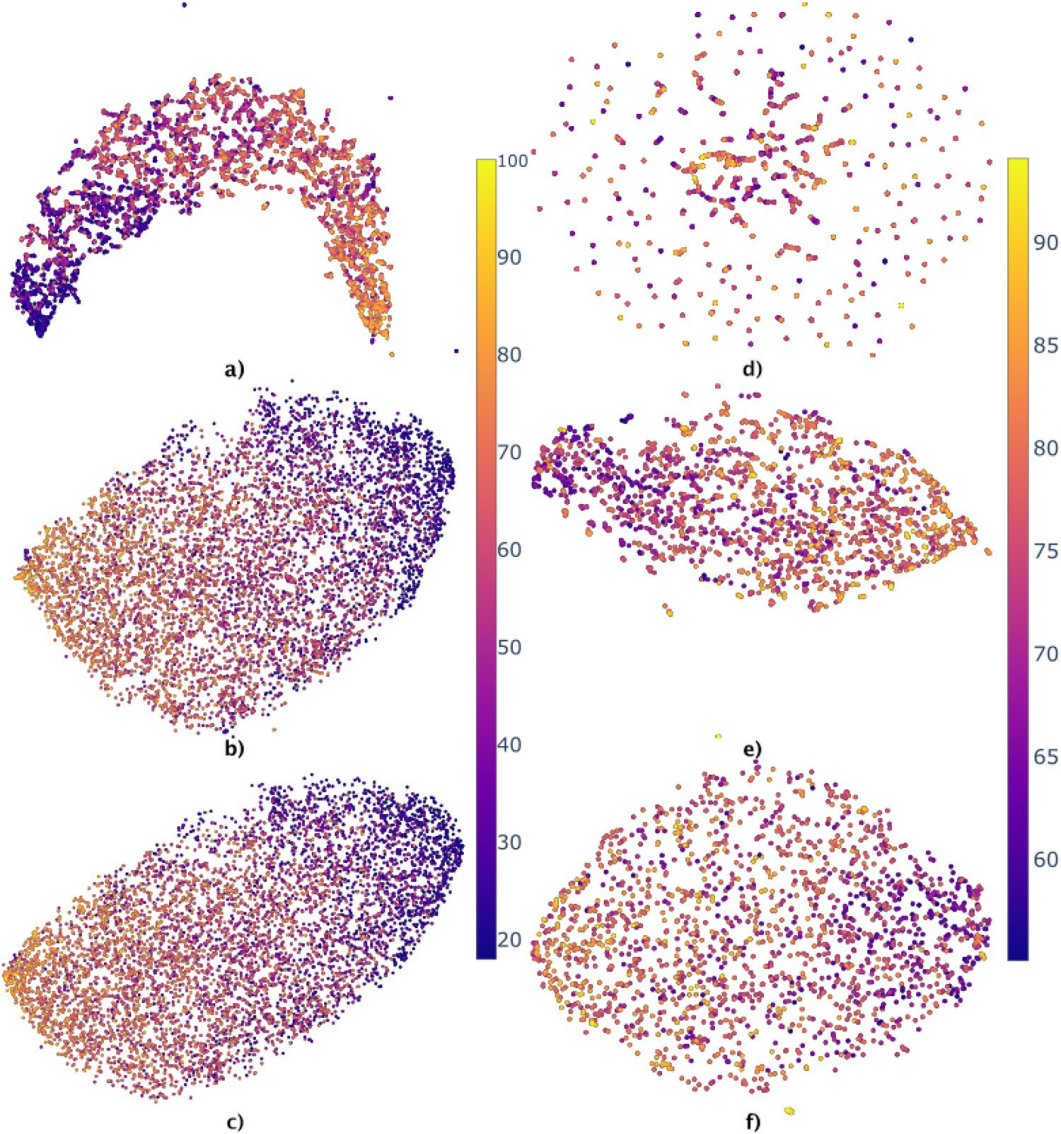
UMAP embeddings of the NOMIS (left figures) database and ADNI database (right figures) using three different values of n_neighbors. The age (years) of the participants is color coded, from 18 to 100 for NOMIS and 55–97 for ADNI. The parameter min_dist = 0 for all embeddings. (**a**) and (**d**) Has n_neighbors = 3, (**b**) and (**e**) has n_neighbors = 20 and (**c**) and (**f**) has n_neighbors = 100. Note that axes have been scaled to fit every point in the frame.

**Figure 4:**
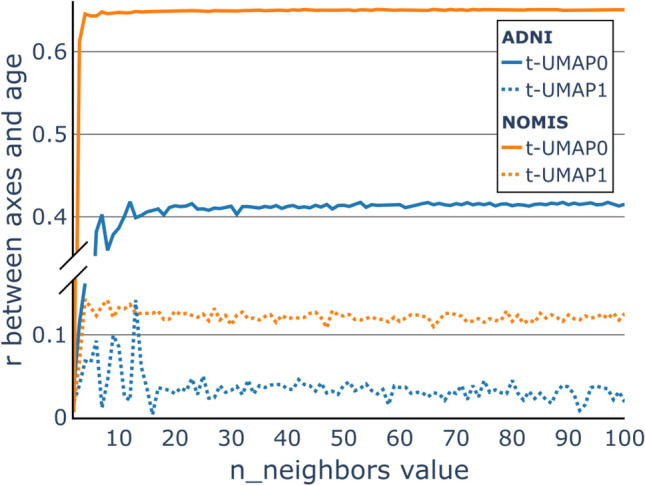
The choice of hyperparameter. The Pearson correlation (*r*) between the values on the t-UMAP0 axis (full line), the values on the t-UMAP1 axis (dashed line) and the age of corresponding participants in the NOMIS dataset (orange) and ADNI dataset (blue). The correlations are plotted against the value of the hyperparameter n_neighbors used to generate the t-UMAP embedding. This allows us to find the value where the correlation is greater.

Using UMAP on the standardized NOMIS and ADNI data, we explored different embedding possibilities as a function of n_neighbors values (Fig. 3). For the NOMIS dataset, each embedding encompasses a strong correlation between the first (UMAP0) axis and age, as shown by the color gradient. However, for high n_neighbors values, changes in the embeddings were less prominent. For instance, Fig. 4 shows that the correlation between t-UMAP0 and age varies only slightly after n_neighbors = 5. On the other hand, for the ADNI embedding, changes were much more prominent. By changing n_neighbors, points were more and more uniformly distributed along the space. Moreover, the participants’ longitudinal scans were closely grouped up in embeddings with lower n_neighbors ( Fig. 3d and e). This result reflects the choice of min_dist=0 since it makes feature vectors resembling each others as close as possible in the UMAP space. On the other hand, age was not well characterized in these models with n_neighbors=3 showing no signs of age-related gradient and n_neighbors $>=$ 20 only displaying clear differences between the extremities.

To select the embedding for our trajectory analysis, we computed the correlation between t-UMAP0 (full line) and t-UMAP1 (dashed line) with the age of subjects from NOMIS (orange) and ADNI (blue) when varying the parameter n_neighbors (Fig. 4). For the NOMIS model, t-UMAP0 at low values of n_neighbors, the correlation steeply increases before quickly converging towards an asymptote. The correlation between age and t-UMAP1 also seems to converge to an asymptotic value albeit in a more noisy fashion. Hence, to choose the optimal n_neighbors value, we wanted to maximize the correlation between t-UMAP0 and age, while minimizing the one between t-UMAP1 and age. Optimal correlations were contained between n_neighbors=20 and n_neighbors=47, meaning that differences were most likely due to random noise. Therefore, to preserve computer resources and the local structure of the underlying manifold, we chose n_neighbors=20.

For the ADNI model, the models were not strongly correlated with age with a maximum *r* just over 0.4 for t-UMAP0 and around 0.05 for t-UMAP1. Even though these models do not seem to be related to biological age, we chose n_neighbors=16 since the correlation with t-UMAP1 is at its minimum and starting to plateau for t-UMAP0.

We also tested higher dimensional embeddings. Using a 3-dimensional embedding with n_neighbors=20, we computed the explained variance of each dimension to quantify the usefulness of each dimension. The first dimension explained 95.91%, the second 2.93% and the third 1.16% of the variance. We felt therefore justified in working with only the first two dimensions.

### Reorientation of UMAP embedding along its principal axes and correlations

**Figure 5:**
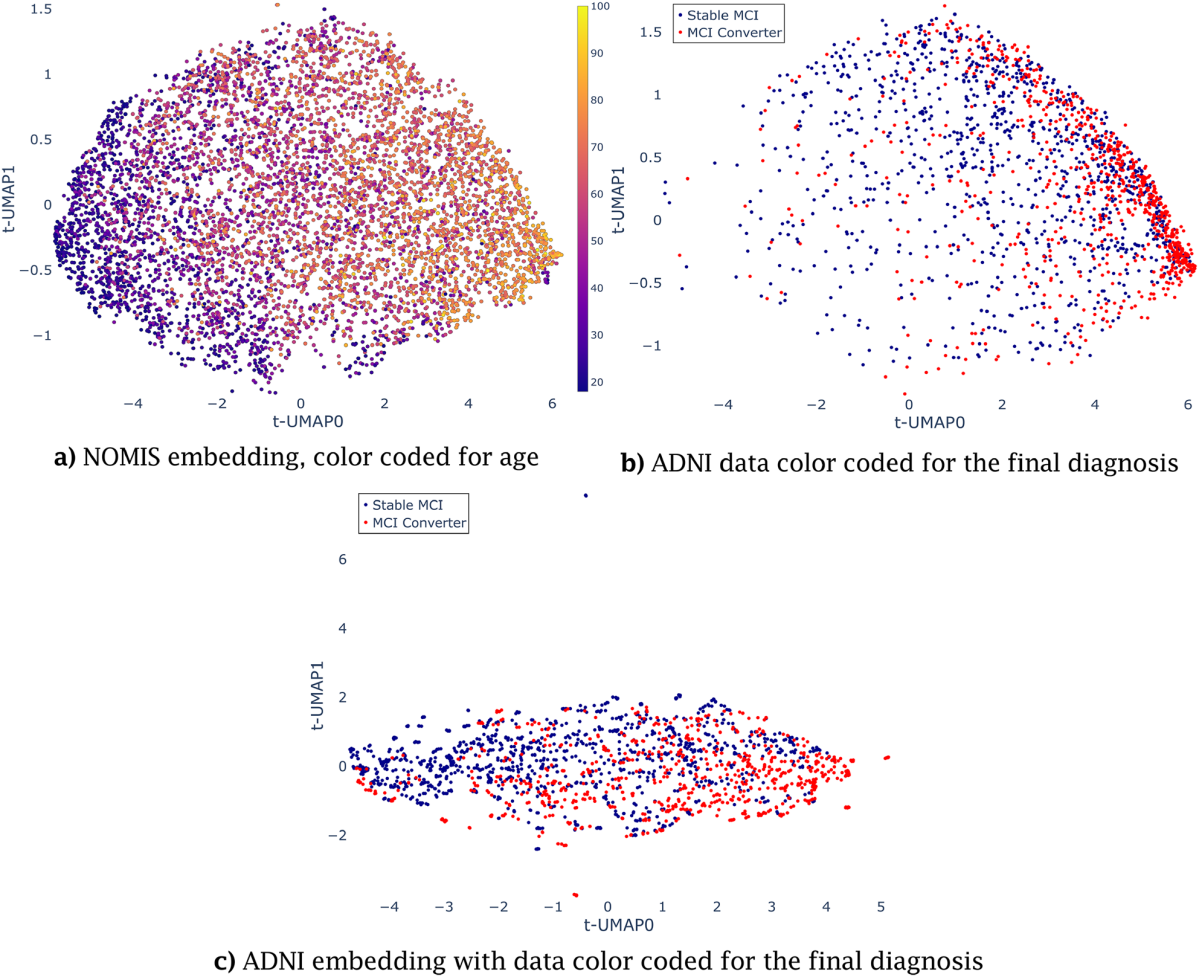
Transformed UMAP embedding and fitted ADNI data. A linear transformation is applied on the UMAP embedding with n_neighbors = 20 and the ADNI embedding with n_neighbors = 16 to align the principal axes with UMAP0 and UMAP1. In (**a**), the NOMIS database (embedding data) with participants’ chronological age (years) at scan color-coded using the color scale displayed on the right. In (**b**), participants from the ADNI database were projected in the NOMIS-defined UMAP embedding. Subject’s final diagnosis was used to color the points on (**b**). One notices how the older ADNI participants (age at entry > 65 y.o.) preferentially concentrate along the positive t-UMAP0 direction, congruent with its correlation with age in the NOMIS dataset. In (**c**), subjects from ADNI are reduced using UMAP and color coded by the final diagnosis.

In our first experiment, once the embedding’s axes were aligned with the principal axes, we noticed that participants’ ages correlated with the transformed main UMAP axis (t-UMAP0; $r = 0.65$) (Fig. 5). It has to be noted that chronological age was not used as an input in the UMAP transformation and this is therefore an emergent property of the embedding. There were six brain regions that were heavily negatively correlated with t-UMAP0 : superior frontal gyrus ($r = -\,0.88$), supramarginal gyrus ($r = -\,0.85$), inferior parietal gyrus ($r = -\,0.83$), rostral middle frontal gyrus ($r = -\,0.83$), caudal middle frontal gyrus ($r = -\,0.82$) and superior temporal gyrus ($r = -\,0.81$). As for t-UMAP1, the peri-calcarine ($r = 0.55$), the cuneus ($r = 0.48$) and the lingual gyrus ($r = 0.47$) were the three regions with the highest correlation. One notices in Fig. 5 how the older ADNI participants (age at entry > 65 y.o.) preferentially concentrate along the positive t-UMAP0 direction, congruent with its correlation with age in the NOMIS dataset.

For the ADNI embedding, Fig. 5**c)** shows different patterns. First, the AD population is once again concentrated to the right, but in a less aggregated manner, implying that although the AD population moves towards the right of the figure, there are no clear attraction points, when compared with the NOMIS embedding. Again, the MCI-MCI are mostly uniformly distributed over the space. The data does not form well-defined clusters, but interestingly they tend to group up in small aggregates of less than 10 points that can look like a single point on Fig. 5**c)** (for example the singular point around (0,7) that is in reality 6 points). The data in these aggregates usually come from the same patient. Interestingly, the correlations for the ADNI embedding are less significant. Most correlations were smaller than previously observed with the four most correlated brain regions being supramarginal gyrus ($r = -0.81$), superior temporal gyrus ($r = -0.76$), superior frontal gyrus ($r = -0.75$) and middle temporal gyrus ($r = -0.74$). The age is no longer correlated ($r = 0.4$). For t-UMAP1, only the entorhinal cortex stands out ($r = 0.54$), with the other regions being mostly uncorrelated ($0.3> r > -0.3$) and the same is true for the age ($r = 0.003$).

For further information, consult Supplementary Tables S1–S4 for the full extent of the correlations between cortical regions and t-UMAP0 and t-UMAP1.

### Trajectories

**Figure 6:**
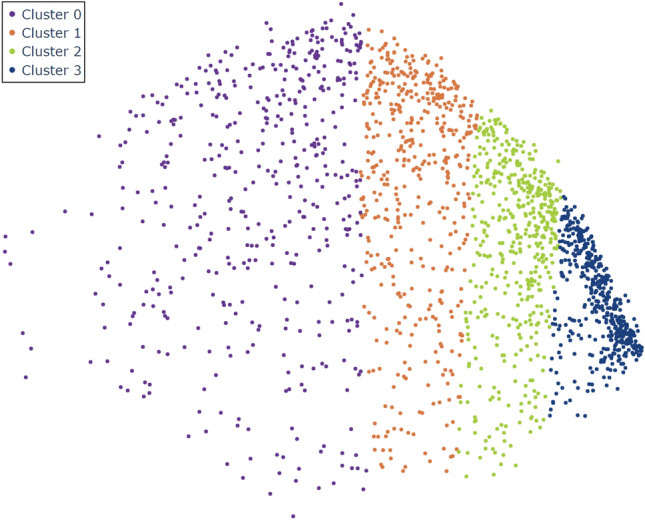
Clustering of the ADNI embedding with same size *k*-means. Dimensional reduction of the ADNI database using a linearly transformed UMAP model trained on the NOMIS database using n_neighbors = 20 and min_dist = 0. The four colors represent the clusters made from the positional values, using a variation of the *k*-means algorithm with n_clusters = 4. For simplicity’s sake, the axes are not displayed since they are the same as Fig. 5.

**Figure 7:**
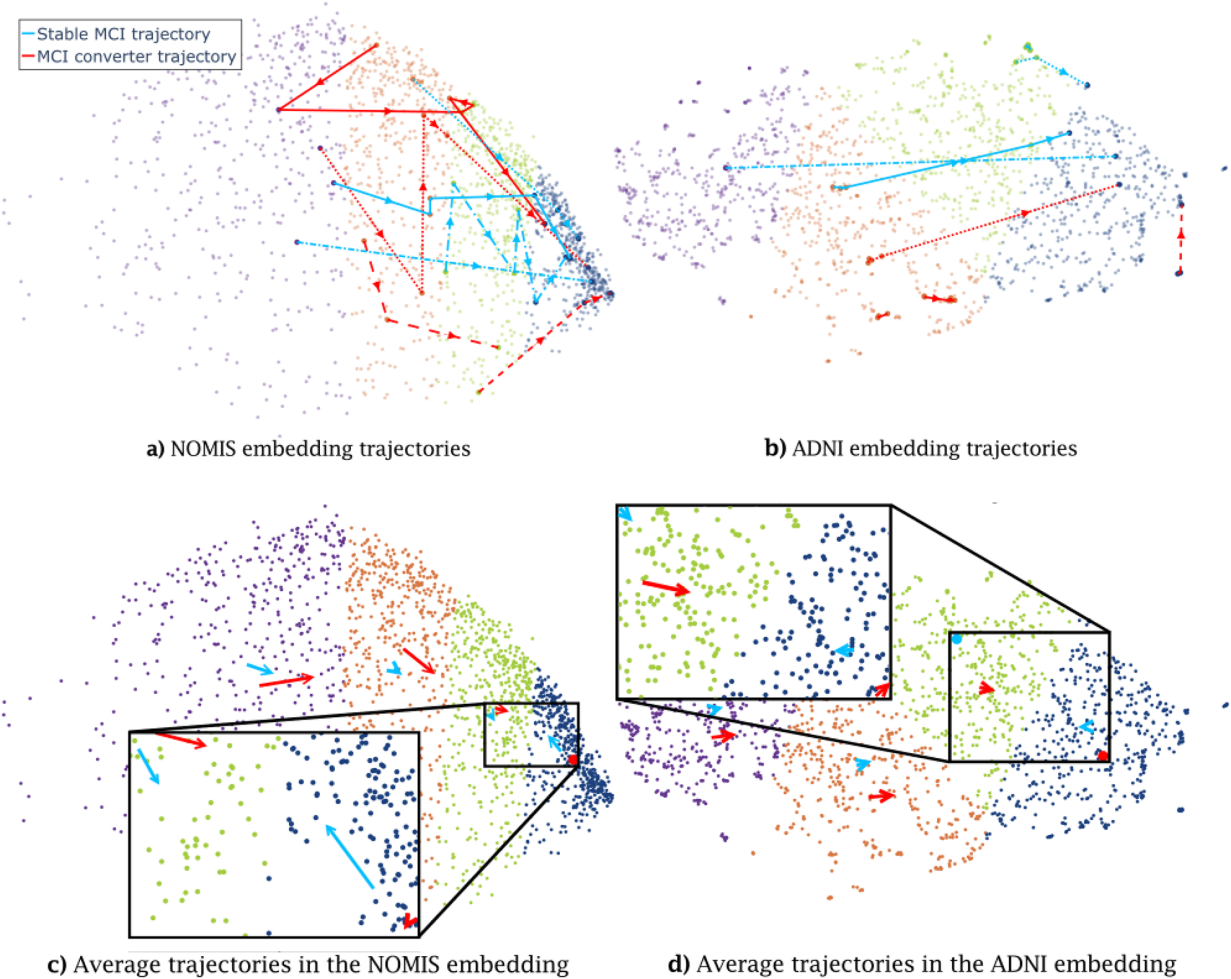
Individual and average trajectories for MCI-MCI and MCI-AD subjects for the NOMIS (left) and ADNI (right) embeddings. On top, the individual trajectory of four MCI-MCI and four MCI-AD patients in the NOMIS embedding (**a**) and the ADNI embedding (**b**). Different dashed lines are used to differentiate between different subjects, within the same diagnosis, and the arrows point in the direction of time. The same patients are shown in both figures. (**c**) and (**d**) Shows the average trajectories for both types of trajectories. Some vectors are represented by a point because the trajectory is too short to be represented by an arrow. (**b**) and (**d**) have had their t-UMAP1 axis cropped to exclude extreme values and visualize trajectories seamlessly.

In our first experiment, to get the trajectory of each individual, we first separated the ADNI dataset into the training data, projected it in the NOMIS t-UMAP space, and separated this embedding into clusters. In Figs. 6 and 7, we used the same_size cluster algorithm defined in the section *Clustering* with n_clusters=4.

For illustrative purpose, we show in Fig. 7 how different participants move through the embedding with time (a “trajectory”). ADNI participants tend to move in a positive direction along t-UMAP0, i.e. from clusters on the left (clusters 0 and 1) to those on the right (clusters 2 and 3) as they age. Some subjects have a direct trajectory to the right-most cluster (solid blue line in Fig. 7a), while others have a more convoluted path (dotted red line in Fig. 7a). On the other hand, some participants move from right to left (not displayed on 7, see 8), in the negative t-UMAP0 direction.

For the ADNI embedding (Fig. 7b), trajectories are less chaotic and most move in a straight direction. Some trajectories, like the full red line, still loop around and some others have less linear paths, but they are much smaller in scale as compared to the linear trajectories. Moreover, some trajectories are much shorter, which is reflected by the fact that the same patient’s scan are often in the same neighborhood.

Figure 7c demonstrates the trends for the average MCI-MCI and MCI-AD groups. Notably, MCI-AD subjects whose trajectory initiates in cluster 3 tend to remain in this cluster, while MCI-MCIs move back towards cluster 2. Also, MCI participants whose trajectory initiates in clusters 1 and 2 display shorter movement towards the right for MCI-MCIs and a greater movement in this direction for AD. Thus, there seems to be an attraction towards a center of mass in cluster 3 for ADs, and in between clusters 1 and 2 for MCI-MCIs.

For Fig. 7d, the average trajectories are overall significantly shorter than those from the NOMIS embedding. We see that MCI-MCI average trajectories are always shorter than MCI-AD’s (excluding the final cluster) and AD trajectories do not exert significant movement in cluster 0 and 1, but all averages are still moving on the t-UMAP0 axis in a positive direction. This can again be explained by the fact that many patient’s scans are grouped closely together, making the averages shorter also. The last cluster seems to again attract AD subjects, but MCI-MCI subjects do not move significantly away from it for this embedding.

**Figure 8:**
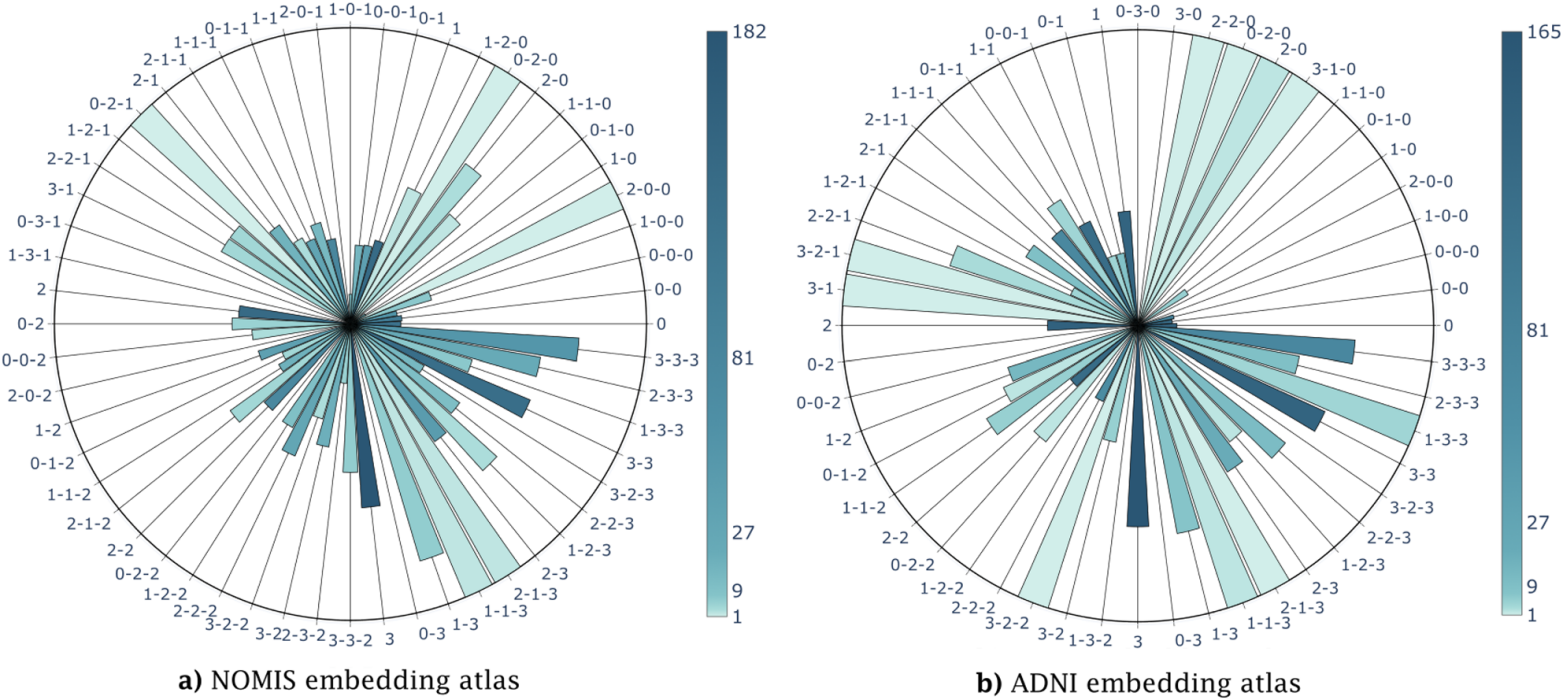
Probabilistic atlas. The distribution of the proportion of AD subjects in each trajectory of length 1–3, color coded for the population in each trajectory. For the sake of visibility, unpopulated trajectories are not displayed. These charts are obtained by counting the number of MCI-MCI and MCI-AD participants in each trajectories. The full bars represent trajectories containing only MCI-AD participants while empty bars have only MCI-MCI participants. (**a**) Is the atlas for the NOMIS embedding and (**b**) the atlas for the ADNI embedding.

Figure 8 displays the probabilistic atlas in its entirety. Using the embedding and clusters from Fig. 6, we computed the proportion of individuals having converted to AD in each possible trajectory of length 1, 2 and 3, using participants’ last *l* data points. This allows us to make some further observations on the trajectories. First of all, in Fig. 8a there seems to be a relationship between the last cluster being visited and prognosis. For instance, trajectories 0 ; 0-0 ; 0-0-0 have a very low probability of developing AD ($< 18\%$), while trajectories 3 ; 3-3 ; 3-3-3 have a probability between $62\%$ and $77\%$ to be converting MCIs, with intermediate clusters displaying a progressive relation. Secondly, out of the 56 trajectories with at least one subject, 30 were monotonically increasing or stable (i.e. moving from lower to higher cluster ID or staying in the same cluster). These seem to follow the previous phenomena related to aging and eventual conversion to AD. The second half of trajectories (n = 26) were more random. However, the population distribution for these trajectories have a median of three subjects; while the five trajectories with 10 or more subjects all had an associated conversion probability of under $42\%$. Thus, they seem more representative of either noisy data or morphological variability without association to conversion. Moreover, trajectories that have a point in cluster 3 without finishing in it have all probabilities $\le 50\%$ and are more populated than most of the abnormal trajectories ; with a median at 4, a mean at 6.6. This shows that these trajectories are more often associated with MCI even though this cluster is by itself more associated with the AD. Finally, there are a number of outlier quadrants with either a probability of zero or one, due to a limited sample size (1–3 subjects). Figure 8b shows a similar pattern. Indeed, we see again that patients in the last cluster have a greater probability of developing AD with this probability augmenting the longer the stay (68–$74\%$) while patients in the first cluster have the lowest probability associated ($<17\%$). On the other hand, the in-between clusters behave differently. Subjects ending in cluster 1 have a higher probability (38–$41\%$) than what was observed in the NOMIS embedding and cluster 2 patients have a lower probability (28–$31\%$) than cluster 1.

### ROC curves

**Figure 9:**
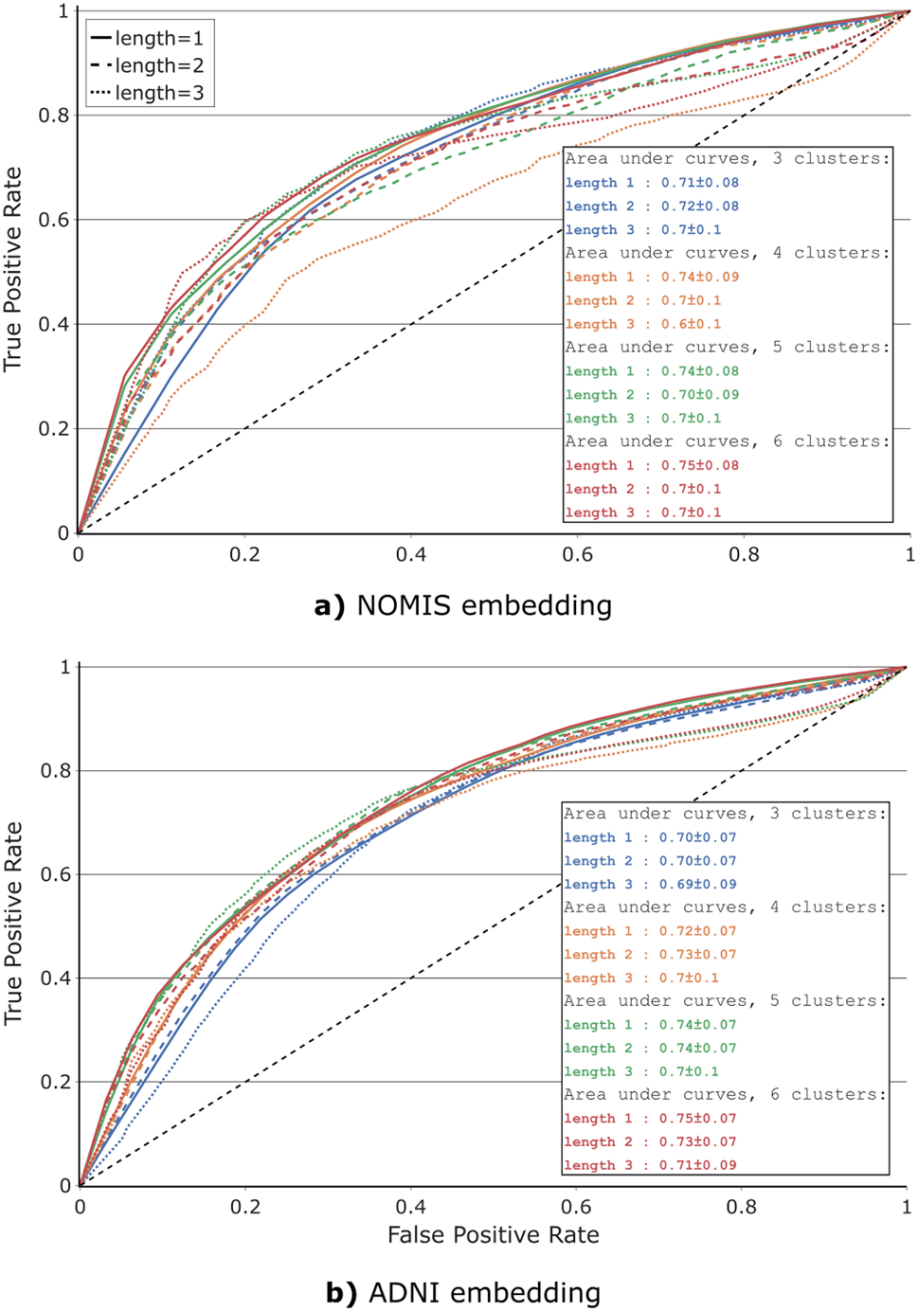
ROC curves of 4 different clustering of the transformed UMAP embeddings (with 3, 4, 5, and 6 clusters). The ROC curves are generated from the classification between MCI-MCI and MCI-AD trajectories probabilities from the probabilistic atlas (Fig. 8). For each clustering, we verify the effect of trajectory length, the last *l* longitudinal time points of a participant; see Fig. 2. (**a**) shows the ROC curves obtained using the NOMIS embedding, while (**b**) is made using the ADNI embedding. Both figures use the same cross-validation set (subset of ADNI). For each clustering, predictions are made using trajectory length of 1, 2, and 3 last time points. The dashed black line represents the diagonal with an area under curve (AUC) of 0.5 for reference. The AUC of each ROC curve is displayed in the table at the bottom right of the figures. This value is obtained by averaging the AUC from the 10-fold resamplings of the train/validation subsets and the value following the ± is the standard deviation of this distribution. Moreover, the ROC curves displayed are obtained by averaging the ROC curves from the same 10-fold resamplings.

We computed the AUC of our ROC curves as an indication of the efficiency of our probabilistic atlas at classifying the test group. We first randomly divided ADNI into a test set (10% of every MCI-MCI and MCI-AD), space set (40%) and a set for the validation process (50%). The latter was then separated into a training group, containing 45% of every MCI-MCI and 45% of every MCI-AD participant, which we used to compute the probabilistic atlas for trajectories of length 1, 2 and 3, with the previously mentioned dimensional reduction method and clustering method. 5% of data left was used to validate the accuracy of our method. Varying the threshold probability value at which the participant was deemed to convert allowed us to generate ROC curves shown on Fig. 9.

The training and validation sets were resampled using 10-fold cross-validation which is repeated 10 times with different random states. The average and standard deviations for this AUC distribution were calculated and are displayed as an insert in Fig. 9. Accuracy grows as a function of n_clusters for a given length, with the most accurate models at n_clusters=6 for the NOMIS embedding and ADNI embedding, but high variance on length 3 makes this relation difficult to confirm. The algorithm was also tested on 7 and 8 clusters, but the AUC plateaued for those. Alongside higher standard deviations caused by the increasing number of empty trajectories (Supplementary Figs. S5 and S6), these models were excluded for simplicity.

Finally, the test set was applied on the NOMIS and ADNI model that yielded the best AUC during validation. From those we got a final accuracy of 0.72 for the NOMIS embedding with 6 clusters and trajectory length of 3 and 0.80 for the ADNI embedding with 4 clusters and trajectory length of 3.

## Discussion

Our goal in the present work was to investigate low-dimensional representations of aging trajectories in order to identify differences between trajectories related to stable cognition and decline due to probable AD. We wanted to develop a method that has a good predictive accuracy, but at the same time, remains interpretable. To do so, we first extracted a vector of cortical thicknesses of different brain regions using FreeSurfer. We then further reduced the dimensionality of the data using the nonlinear method UMAP and linearly transformed the UMAP embedding while maximizing the correlation of the main axis with age. We then investigated the different aging trajectories in this space and found significant differences between MCI-MCI and MCI-AD trajectories. We estimated that our novel approach could yield a good predictive accuracy, with a maximum AUC of about 0.72 on the NOMIS embedding and 0.8 on ADNI embedding. We now discuss the novelty of our approach, its limitations and possible future research directions.

### UMAP as a nonlinear embedding technique

UMAP was chosen over PCA since the former provided a more compact and homogeneous embedding of the data, with a lesser standard deviation in both the *x* and *y* axes, which is advantageous when dividing the space into clusters of similar size. The nonlinearity of UMAP is advantageous as it represents more accurately cortical thickness atrophy happening with age, while preserving both local and global features of the original high-dimensional dataset. The optimization of UMAP parameters required us to fix min_dist to 0. This means that two MRIs yielding the same image will occupy the same position in the t-UMAP space. This is useful since it will induce a positive distance between two points only if they are different, and thus a participant’s trajectory is not artificially modified. Furthermore, two similar points are more likely to be in the same cluster. Then, for the parameter n_neighbors, Fig. 3 showed the different embeddings given a value of the parameter. As explained in the UMAP paper^30^, lower values make the embedding locally connected while higher values make it globally connected, meaning that global structures in the data are represented over local ones. The figure represents this by showing the softening of internal structures for high n_neighbors values. Indeed, for the lowest value of n_neighbors, the embedding from NOMIS takes a very different form. With low values, local resemblance between members of the NOMIS database are put forth while the more n_neighbors is high and the more the embedding starts looking alike : points are uniformly distributed inside an ellipsoid. This is even more apparent for the ADNI embeddings where, on low values, the data is grouped in tightly packed clusters to the point of resembling a single data point in some case. Increasing n_neighbors also causes this embedding to take an elliptical form, while conserving a level of local connectivity. This connectivity comes most likely from the fact that ADNI is a longitudinal database as opposed to NOMIS. Since the data is standardized, a scan should be located in the vicinity of its subject’s other scans in the UMAP space, unless a significant change happened in the morphometry. In the embedding, this is reflected by a locally connected plot where each subject’s scans are often grouped together in the space, even with high n_neighbors. For the current paper, we prioritize global structures since embeddings with lower n_neighbors are less suitable for the same reason PCA was not. Moreover, we chose the correlation between t-UMAP0 and subject’s age as a metric of how much the embedding encompasses the aging process. Figure 4 shows that this value is optimized from n_neighbors = 20 for the NOMIS embedding and n_neighbors = 16 for the ADNI embedding. In light of this, we chose the embedding with n_neighbors at 20 and 16 since it offered a good compromise between local and global structures and the correlation with age is optimized for all axes (Fig. 4). Finally, before settling on a two-dimensional representative space, we verified that a higher-order embedding would not better fit the data. The explained variance of the third dimension (1.16%) showed that the first two dimensions were sufficient to represent our datasets.

In hindsight, the correlation with age might not be the most accurate metric for choosing n_neighbors. Although it makes sense for the point of view of having an embedding representing aging, the fact that the ADNI embedding has very low correlations with age and yet manages to obtain better predictive accuracy shows that this metric doesn’t paint a full picture of the embedding’s predictive power. Future studies should either find a relevant measure to evaluate the embedding, or if computational power is not a problem, include n_neighbors as a parameter of the cross-validation process.

### Age dependency

Applying a linear transformation on the embedding in order to align the principal axes with the UMAP0 and UMAP1 axes yielded a new space where the t-UMAP0 axis correlated the most with the age. Indeed, the age gradient on Fig. 5 seems to progress parallel to the axis with younger individuals are located on the left of the figure while older ones are on the right. The t-UMAP0 axis can be interpreted as a participant’s “brain age”; the fact that some younger participants had a strong “positive” brain age is indicative of individual variability. Note however that *FreeSurfer* seems to introduce significant noise in morphometric measurements, which causes highly variable trajectories for some individuals.

In this paper, we specifically chose to use a dataset of healthy individuals with a wide age range to train the UMAP embedding. This was done with the idea that the embedding would encompass the normal aging process which would make it easier to differentiate AD trajectories down the line. In a way, it is what we were able to do since the embedding correlates with the brain age of subjects. Interestingly enough, the data is relatively uniformly distributed in the embedding and no clearly defined clusters are present. This shows again that UMAP wasn’t able to find defined characteristics in the data and so every subject is represented in a spectrum of the aging process. Moreover, we chose to use no prior to select the features to be reduced. This again goes with the idea we wanted the embedding to represent the aging process of the brain. For the specific purpose of predicting decline, we could have used *Dickerson* et al.’s cortical AD signature^7^ to select specific AD related regions, which would have helped increase the sensitivity of our model and hence its predictive power. On that note, the inferior parietal and superior frontal gyrus are both heavily correlated with the t-UMAP0 axis and have been identified as regions within the AD signature^7^. On the other hand, the ADNI embedding doesn’t express similar correlations. The correlations are all lower as compared to the NOMIS embedding (− 0.81 versus − 0.76 for the most negatively correlated regions), with only the superior frontal gyrus, supramarginal gyrus, and superior temporal gyrus being still strongly correlated with t-UMAP0. The age dependency completely disappeared for the ADNI embedding both for t-UMAP0 and t-UMAP1. It is likely that, since ADNI’s age range is restricted, so is the cortical thickness loss due to aging. A possible other explanation comes from the fact that MCI and AD-related aging should not affect cortical thickness the same way as non-pathological aging. This way, the subject’s state shouldn’t be correlated with its age.

### Trajectory definition and analyses

From Fig. 7c and d it is clear that MCI-MCI trajectories are different from MCI-AD. Both trajectory types have different attracting points and different averages, although it could be construed that most of these differences between trajectory types could be explained by the differences in the samples represented in each type. The ADNI embedding displays similar properties, as exemplified by differences in the average trajectories between MCI-MCI and MCI-AD in Fig. 7d. For this embedding, since none of the axis are correlated with age, the differences between both groups can only be explained by an underlying property of the embedding. On the other hand, the average trajectories have a smaller norm overall. Scans coming from a same individual are usually grouped together in the same cluster.

### Clusters

Some interesting patterns emerge in the atlas shown in Fig. 8a. First of all, there is a strong relationship between the final cluster and AD diagnosis. Indeed, trajectories with the most subjects show a trend where subjects with a final scan in cluster 0 have a low ($\sim$ 17%) probability of developing AD and the probability goes up with the index of the final cluster : cluster 1 $\sim$30%, cluster 2 $\sim$38% and cluster 3 $\sim$62%. Moreover, the longer a subject stays in the cluster the stronger is this trend. For example, the 3-step trajectory “0-0-0” has a similar probability than its homologous 1-step trajectory “0”, while “1-1-1” shows a $\sim$2% increase in probability when compared to “1”, “2-2-2” a $\sim$10% increase when compared to “2” and “3-3-3” a $\sim$15% increase when compared to “3”. Since age is distributed similarly between AD and MCI-MCI subjects, there seems to be a clear relationship between cluster 3 and the diagnosis of AD. Moreover, trajectories with an intermediary data point in cluster 3 and another in cluster 0, 1 or 2 afterwards, have a significantly lower probability to describe AD. This phenomenon is directly related to our observations that MCI-MCI subjects in cluster 3 go towards the other clusters on average, while AD subjects stay in cluster 3. From this figure we can then conclude the clusters and trajectories are correlated with certain diagnosis.

The ADNI embedding in Fig. 8b shows very similar trends. We already know that the first and last cluster are respectively associated with the lowest and highest AD probability. The behavior at the center is much different for the ADNI embedding, since the probability doesn’t seem to augment the closer you move to the right of the figure. Indeed we saw that cluster 1 (38–41%) has a higher probability than cluster 2 (28–31%). This discrepancy can be explained by the fact that clusters 1 and 2 are overlapping on the t-UMAP0 (see Fig. 7) as compared to the NOMIS embedding where clusters mostly encompass a different range on the axis. Additionally, from Fig. 5c) we observed that AD subjects seems to prefer lower values of the t-UMAP1 axis, which also supports the fact that cluster 1 is associated with a higher probability developing AD. From this, both embeddings have prediction potential based on their clustering.

The predictive analysis shown in Fig. 9 corroborates the previous observations. The AUC shows that trajectories, based on cortical thickness only, are able to classify subjects between MCI-MCIs and MCI-ADs in more than 72% of cases for the best models (6 clusters) on the test set. Even though the AUC is higher on average for trajectories of length 1 on the validation set, the models with the highest predictive power was, for both embedding with length of 3. For every model of the NOMIS embedding, the essential information necessary for classification seems to be located in the last cluster of the trajectory, since longer trajectory length reduces the accuracy and enhances the standard deviation on the validation set. For the ADNI embedding the predictive power on the validation set is more divided between length of one and two, with the model with four clusters having a higher AUC with length of two. On the test set, the ADNI embedding manages to outperform the NOMIS embedding with a respective AUC of 0.80 and 0.72. The embedding trained directly on the ADNI space set seems better suited for predicting decline than the NOMIS embedding, even though the ladder has a strong correlation with participants age. The hypothesis that an embedding representing normal aging would help differentiate participants on the AD spectrum is then incorrect.

## Conclusion

This study explored the visualization and statistical analysis of morphometric aging trajectories using UMAP, a novel dimensionality reduction technique, for subjects with AD or a MCI. We demonstrated the potential advantages of using this approach. We showed that using this method leads to an embedding space in which subjects exhibit trajectories between clusters that are significantly different depending on their remaining cognitively stable or declining to AD. Our results thus indicate that UMAP is a new and effective tool that not only can visualize aging trajectories, but also accurately differentiate between MCI-AD and MCI-MCI trajectories. To further improve the accuracy of the model, it would be of interest to perform a UMAP-based analysis using only the thickness of cortical regions related to AD^7,35,47^ and other data related to AD biomarkers such as amyloid beta concentration^48,49^, cognitive score^50^ and cognitive reserve^51^.

### Supplementary Information

Supplementary Information.

## Data Availability

We are not the owners of the data used in this study, but all the databases are publicly available upon request. The links to the different databases and the instructions to obtain the datasets, can be found here : Autism Brain Imaging Data Exchange (ABIDE): https://fcon_1000.projects.nitrc.org/indi/abide/. Alzheimer’s Disease Neuroimaging Initiative (ADNI): http://adni.loni.usc.edu/. Australian Imaging Biomarkers and Lifestyle flagship study of ageing (AIBL) : https://aibl.csiro.au/. Berlin Mind and Brain (Margulies, Villringer) CoRR sample (BMB): https://fcon_1000.projects.nitrc.org/indi/CoRR/html/bmb_1.html. Cambridge Centre for Ageing and Neuroscience (CamCAN): https://www.mrc-cbu.cam.ac.uk/datasets/camcan/. Center of Biomedical Research Excellence (COBRE): https://fcon_1000.projects.nitrc.org/indi/retro/cobre.html. Cleveland Clinic (Cleveland CCF): https://fcon_1000.projects.nitrc.org/indi/retro/ClevelandCCF.html. Consortium for the Early Identification of Alzheimer’s Disease (CIMA-Q): https://www.cima-q.ca. Dallas Lifespan Brain Study (DLBS): https://fcon_1000.projects.nitrc.org/indi/retro/dlbs.html. FIND lab sample: https://fcon_1000.projects.nitrc.org/indi/retro/find_stanford.html. Functional Biomedical Informatics Research Network (FBIRN): https://www.birncommunity.org/resources/data/. Lifespan Human Connectome Project in Aging (HCP-Aging): http://dx.doi.org/10.15154/1520138. International Consortium for Brain Mapping (ICBM): https://ida.loni.usc.edu/login.jsp?project=ICBM. Information eXtraction from Images (IXI): http://brain-development.org/ixi-dataset/. F.M. Kirby Research Center neuroimaging reproducibility data (KIRBY-21): https://www.nitrc.org/projects/multimodal. Minimal Interval Resonance Imaging in Alzheimer’s Disease (MIRIAD): http://miriad.drc.ion.ucl.ac.uk. National Alzheimer’s Coordinating Center (NACC): https://www.alz.washington.edu/. National Database for Autism Research (NDAR): http://nda.nih.gov. Nathan Kline Institute Rockland (NKI-R) sample (NKI-RS): https://fcon_1000.projects.nitrc.org/indi/pro/nki.html. NKI-R enhanced Sample (NKI-RES): https://fcon_1000.projects.nitrc.org/indi/enhanced/. Open access series of imaging studies (OASIS): https://www.oasis-brains.org/. POWER: https://fcon_1000.projects.nitrc.org/indi/retro/Power2012.html. Parkinson’s Progression Markers Initiative (PPMI): https://www.ppmi-info.org. Southwest University Adult Lifespan Dataset (SALD): https://fcon_1000.projects.nitrc.org/indi/retro/sald.html. University of Wisconsin, Madison (Birn, Prabhakaran, Meyerand) CoRR sample (UWM): https://fcon_1000.projects.nitrc.org/indi/CoRR/html/uwm_1.html. Wayne State EF Dataset: https://fcon_1000.projects.nitrc.org/indi/retro/wayne_EF.html. Yale Low-Resolution Controls Dataset: https://fcon_1000.projects.nitrc.org/indi/retro/yale_lowres.html.
