# Author Correction: Predicting cognitive decline in a low-dimensional representation of brain morphology

**DOI:** 10.1038/s41598-023-46972-6

**Published:** 2023-11-17

**Authors:** Rémi Lamontagne-Caron, Patrick Desrosiers, Olivier Potvin, Nicolas Doyon, Simon Duchesne

**Affiliations:** 1 Département de médecine, Université Laval, Quebec, QC G1V 0A6 Canada; 2 Centre de recherche CERVO, Quebec, QC G1J 2G3 Canada; 3 Centre interdisciplinaire en modélisation mathématique, Université Laval, Quebec, QC G1V 0A6 Canada; 4 Département de physique, de génie physique et d’optique, Université Laval, Quebec, QC G1V 0A6 Canada; 5 Département de mathématiques et de statistique, Université Laval, Quebec, QC G1V 0A6 Canada; 6 Département de radiologie et médecine nucléaire, Université Laval, Quebec, QC G1V 0A6 Canada

Correction to: *Scientific Reports* https://doi.org/10.1038/s41598-023-43063-4, published online 05 October 2023

The original version of this Article contained an error in the Acknowledgments section, where the link to the list of ADNI investigators was inadvertently omitted.

“Data used in preparation of this article were obtained from the Alzheimer’s Disease Neuroimaging Initiative (ADNI) database (adni.loni.usc.edu). As such, the investigators within the ADNI contributed to the design and implementation of ADNI and/or provided data but did not participate in analysis or writing of this report. A complete listing of ADNI investigators can be found at this link.”

now reads:

“Data used in preparation of this article were obtained from the Alzheimer’s Disease Neuroimaging Initiative (ADNI) database (adni.loni.usc.edu). As such, the investigators within the ADNI contributed to the design and implementation of ADNI and/or provided data but did not participate in analysis or writing of this report. A complete listing of ADNI investigators can be found at this link: http://adni.loni.usc.edu/wp-content/uploads/how_to_apply/ADNI_Acknowledgement_List.pdf.”

The original Article has been corrected.

