# Supplementary material for: Predicting cognitive decline in a low-dimensional representation of brain morphology
Source: Sci Rep. 2023 Oct 5;13:16793. doi: 10.1038/s41598-023-43063-4 (PMC10556003; doi:10.1038/s41598-023-43063-4)
Supplement: Supplementary file 1 — Supplementary Information. [file 41598_2023_43063_MOESM1_ESM.pdf]

Cortical regions	Pearson correlation	Cortical regions	Pearson correlation
superiorfrontal_r	-0.876952	parsorbitalis_r	-0.675987
superiorfrontal_l	-0.875407	lateralorbitofrontal_l	-0.66624
supramarginal_l	-0.853448	posteriorcingulate_l	-0.64067
supramarginal_r	-0.834146	lateralorbitofrontal_r	-0.633283
inferiorparietal_l	-0.829533	posteriorcingulate_r	-0.623178
rostralmiddlefrontal_l	-0.826265	lateraloccipital_l	-0.61807
caudalmiddlefrontal_l	-0.824142	lateraloccipital_r	-0.615145
inferiorparietal_r	-0.820774	fusiform_r	-0.605319
superiortemporal_r	-0.806394	inferiortemporal_r	-0.602292
caudalmiddlefrontal_r	-0.805738	caudalanteriorcingulate_l	-0.58978
rostralmiddlefrontal_r	-0.80231	inferiortemporal_l	-0.586643
superiortemporal_l	-0.801841	transversetemporal_r	-0.574015
parstriangularis_l	-0.793562	rostralanteriorcingulate_l	-0.573386
middletemporal_r	-0.791966	transversetemporal_l	-0.561767
parsopercularis_r	-0.790692	cuneus_l	-0.538545
parstriangularis_r	-0.788787	cuneus_r	-0.535733
middletemporal_l	-0.785394	medialorbitofrontal_r	-0.519666
parsopercularis_l	-0.783172	isthmuscingulate_l	-0.504857
precuneus_l	-0.782538	medialorbitofrontal_l	-0.501384
precuneus_r	-0.771401	isthmuscingulate_r	-0.489114
precentral_l	-0.768512	lingual_l	-0.434558
precentral_r	-0.763119	rostralanteriorcingulate_r	-0.423809
superiorparietal_l	-0.740365	lingual_r	-0.418618
superiorparietal_r	-0.73864	caudalanteriorcingulate_r	-0.407113
postcentral_l	-0.716919	pericalcarine_r	-0.343675
insula_r	-0.711307	parahippocampal_r	-0.330147
paracentral_r	-0.699701	pericalcarine_l	-0.318649
postcentral_r	-0.69633	parahippocampal_l	-0.304155
paracentral_l	-0.689912	entorhinal_l	-0.21535
parsorbitalis_l	-0.686529	entorhinal_r	-0.18102
insula_l	-0.686017	age	0.649034
fusiform_l	-0.682929		

Supplementary Table S1. t-UMAP0's correlation with cortical regions and age. The Pearson correlation between the t-UMAP0 axis and the thickness of the different cortical regions used in the dimensional reduction and the subjects' age. These values were obtained with the UMAP parameters : `n_neighbors` = 20, `min_dist` = 0 and `random_state` = 42.

Cortical regions	Pearson correlation	Cortical regions	Pearson correlation
pericalcarine_l	0.552909	superiortemporal_r	-0.0364093
pericalcarine_r	0.547725	middletemporal_l	-0.0367981
cuneus_r	0.48084	parstriangularis_l	-0.0373123
lingual_r	0.474045	parsopercularis_l	-0.0509358
lingual_l	0.471035	entorhinal_r	-0.0631519
cuneus_l	0.440937	middletemporal_r	-0.0642912
transversetemporal_l	0.294846	isthmuscingulate_r	-0.0678302
transversetemporal_r	0.274237	rostralmiddlefrontal_l	-0.0854371
lateraloccipital_r	0.266643	entorhinal_l	-0.0916308
postcentral_r	0.264153	parsopercularis_r	-0.0959004
postcentral_l	0.259048	parsorbitalis_l	-0.100174
lateraloccipital_l	0.251781	parstriangularis_r	-0.10494
superiorparietal_r	0.225238	superiorfrontal_l	-0.111455
superiorparietal_l	0.210182	isthmuscingulate_l	-0.111712
precentral_l	0.193447	inferiortemporal_r	-0.118378
precentral_r	0.180491	parsorbitalis_r	-0.144352
paracentral_r	0.165205	superiorfrontal_r	-0.147209
precuneus_r	0.144318	rostralmiddlefrontal_r	-0.165768
paracentral_l	0.137474	insula_r	-0.177589
age	0.128916	insula_l	-0.180815
precuneus_l	0.118249	posteriorcingulate_r	-0.185355
parahippocampal_r	0.078403	posteriorcingulate_l	-0.190291
fusiform_r	0.0779755	inferiortemporal_l	-0.195443
inferiorparietal_r	0.057548	lateralorbitofrontal_l	-0.20209
fusiform_l	0.0571907	medialorbitofrontal_l	-0.224996
parahippocampal_l	0.051324	medialorbitofrontal_r	-0.228779
inferiorparietal_l	0.0422169	caudalanteriorcingulate_l	-0.271913
supramarginal_r	0.0414675	lateralorbitofrontal_r	-0.277769
supramarginal_l	0.030307	caudalanteriorcingulate_r	-0.278458
superiortemporal_l	0.015318	rostralanteriorcingulate_l	-0.289819
caudalmiddlefrontal_l	-0.00400514	rostralanteriorcingulate_r	-0.305279
caudalmiddlefrontal_r	-0.0306759		

Supplementary Table S2. t-UMAP1's correlation with cortical regions and age. The Pearson correlation between the t-UMAP1 axis and the thickness of the different cortical regions used in the dimensional reduction and the subjects' age. These values were obtained with the UMAP parameters : `n_neighbors` = 20, `min_dist` = 0 and `random_state` = 42.

Cortical regions	Pearson correlation	Cortical regions	Pearson correlation
supramarginal_l	-0.805249	paracentral_l	-0.60698
superiortemporal_l	-0.760692	superiorparietal_l	-0.602568
superiorfrontal_r	-0.748668	insula_l	-0.589436
superiorfrontal_l	-0.745961	isthmuscingulate_r	-0.586814
supramarginal_r	-0.745148	lateralorbitofrontal_l	-0.585709
middletemporal_l	-0.73983	lateralorbitofrontal_r	-0.58525
inferiorparietal_r	-0.732523	paracentral_r	-0.582005
middletemporal_r	-0.732462	transversetemporal_l	-0.578652
inferiorparietal_l	-0.730992	isthmuscingulate_l	-0.565313
superiortemporal_r	-0.727618	posteriorcingulate_l	-0.561167
caudalmiddlefrontal_l	-0.715281	transversetemporal_r	-0.555162
rostralmiddlefrontal_l	-0.714318	lingual_l	-0.552015
precentral_l	-0.688223	cuneus_r	-0.5468
fusiform_l	-0.685649	lingual_r	-0.543439
inferiortemporal_l	-0.684909	medialorbitofrontal_r	-0.538125
postcentral_l	-0.679531	parsorbitalis_r	-0.5379
parstriangularis_l	-0.677658	parahippocampal_r	-0.524067
caudalmiddlefrontal_r	-0.674414	parsorbitalis_l	-0.512752
precentral_r	-0.673952	cuneus_l	-0.499179
precuneus_r	-0.669186	posteriorcingulate_r	-0.494424
rostralmiddlefrontal_r	-0.660783	entorhinal_r	-0.489214
precuneus_l	-0.654511	entorhinal_l	-0.480666
inferiortemporal_r	-0.651552	rostralanteriorcingulate_l	-0.461693
postcentral_r	-0.651014	pericalcarine_r	-0.450832
parsopercularis_r	-0.648596	medialorbitofrontal_l	-0.448504
parsopercularis_l	-0.64751	pericalcarine_l	-0.446945
lateraloccipital_l	-0.642215	parahippocampal_l	-0.408416
insula_r	-0.64139	caudalanteriorcingulate_l	-0.398887
fusiform_r	-0.636744	rostralanteriorcingulate_r	-0.364156
lateraloccipital_r	-0.62957	caudalanteriorcingulate_r	-0.223265
parstriangularis_r	-0.618905	age	0.407733
superiorparietal_r	-0.609673		

Supplementary Table S3. t-UMAP0's correlation with cortical regions and age for the ADNI embedding. The Pearson correlation between the t-UMAP0 axis and the thickness of the different cortical regions used in the dimensional reduction and the subjects' age. These values were obtained with the UMAP parameters : `n_neighbors` = 16, `min_dist` = 0 and `random_state` = 42.

entorhinal_l	0.537665	parsopercularis_r	-0.0707972
entorhinal_r	0.518321	posteriorcingulate_l	-0.0769428
parahippocampal_r	0.329262	caudalanteriorcingulate_l	-0.084356
parahippocampal_l	0.328523	parstriangularis_r	-0.104406
inferiortemporal_r	0.213144	cuneus_r	-0.106049
superiortemporal_r	0.200402	transversetemporal_l	-0.106363
superiortemporal_l	0.18822	inferiorparietal_r	-0.108123
inferiortemporal_l	0.183617	supramarginal_r	-0.113103
middletemporal_r	0.15229	superiorfrontal_r	-0.118693
fusiform_r	0.149542	rostralmiddlefrontal_r	-0.121148
insula_l	0.140429	precuneus_r	-0.122006
insula_r	0.131936	rostralmiddlefrontal_l	-0.125883
fusiform_l	0.127449	precentral_r	-0.128905
middletemporal_l	0.123229	cuneus_l	-0.131271
medialorbitofrontal_r	0.0737625	supramarginal_l	-0.132471
rostralanteriorcingulate_l	0.0574475	parsorbitalis_l	-0.138037
lateralorbitofrontal_l	0.0450584	parsopercularis_l	-0.14382
isthmuscingulate_l	0.0210627	posteriorcingulate_r	-0.153789
medialorbitofrontal_l	0.019158	superiorparietal_r	-0.153912
lateralorbitofrontal_r	0.0166662	precuneus_l	-0.155209
pericalcarine_r	0.00715549	caudalanteriorcingulate_r	-0.161414
age	0.00322331	paracentral_l	-0.164732
pericalcarine_l	0.00205908	superiorparietal_l	-0.169337
rostralanteriorcingulate_r	-0.00119598	parstriangularis_l	-0.173961
lingual_l	-0.018495	superiorfrontal_l	-0.173991
isthmuscingulate_r	-0.0297047	caudalmiddlefrontal_l	-0.174653
lateraloccipital_l	-0.0488989	postcentral_r	-0.176028
lateraloccipital_r	-0.0558424	caudalmiddlefrontal_r	-0.180872
transversetemporal_r	-0.0590221	precentral_l	-0.189614
parsorbitalis_r	-0.0665719	postcentral_l	-0.21358
inferiorparietal_l	-0.0684489	paracentral_r	-0.218963
lingual_r	-0.0685776		

Supplementary Table S4. t-UMAP1's correlation with cortical regions and age for the ADNI embedding. The Pearson correlation between the t-UMAP0 axis and the thickness of the different cortical regions used in the dimensional reduction and the subjects' age. These values were obtained with the UMAP parameters : `n_neighbors` = 16, `min_dist` = 0 and `random_state` = 42.

Diagnosis	At Baseline	At Last follow-up
CH	526	477
MCI	828	537
AD	0	340

Supplementary Table S5. The distribution of diagnosis in the ADNI database at baseline and on the last available follow-up. These values were calculated from the raw data obtained.

002_S_0295	002_S_0413	002_S_0559	002_S_0619	002_S_0685	002_S_0729	002_S_0782	002_S_0816
002_S_0938	002_S_0954	002_S_0955	002_S_1018	002_S_1070	002_S_1155	002_S_1261	002_S_1268
002_S_1280	003_S_0907	003_S_0908	003_S_0931	003_S_0981	003_S_1021	003_S_1057	003_S_1059
003_S_1074	003_S_1122	003_S_1257	005_S_0221	005_S_0222	005_S_0223	005_S_0324	005_S_0448
005_S_0546	005_S_0553	005_S_0572	005_S_0602	005_S_0610	005_S_0814	005_S_0929	005_S_1341
006_S_0484	006_S_0498	006_S_0547	006_S_0653	006_S_0675	006_S_0681	006_S_0731	006_S_1130
007_S_0041	007_S_0068	007_S_0070	007_S_0101	007_S_0128	007_S_0249	007_S_0293	007_S_0316
007_S_0344	007_S_0414	007_S_0698	007_S_1206	007_S_1222	007_S_1248	007_S_1304	007_S_1339
009_S_0751	009_S_0842	009_S_0862	009_S_1030	009_S_1199	009_S_1334	009_S_1354	010_S_0067
010_S_0161	010_S_0419	010_S_0420	010_S_0422	010_S_0472	010_S_0786	010_S_0829	010_S_0904
011_S_0002	011_S_0003	011_S_0005	011_S_0008	011_S_0010	011_S_0016	011_S_0021	011_S_0022
011_S_0023	011_S_0053	011_S_0168	011_S_0183	011_S_0241	011_S_0326	011_S_0362	011_S_0856
011_S_0861	011_S_1080	011_S_1282	012_S_0634	012_S_0637	012_S_0689	012_S_0712	012_S_0720
012_S_0803	012_S_0917	012_S_0932	012_S_1009	012_S_1033	012_S_1133	012_S_1165	012_S_1175
012_S_1212	012_S_1292	012_S_1321	013_S_0240	013_S_0325	013_S_0502	013_S_0575	013_S_0592
013_S_0699	013_S_0860	013_S_0996	013_S_1035	013_S_1120	013_S_1161	013_S_1186	013_S_1205
013_S_1275	013_S_1276	014_S_0169	014_S_0328	014_S_0356	014_S_0519	014_S_0520	014_S_0548
014_S_0557	014_S_0558	014_S_0563	014_S_0658	014_S_1095	016_S_0354	016_S_0359	016_S_0538
016_S_0590	016_S_0702	016_S_0769	016_S_0991	016_S_1028	016_S_1092	016_S_1117	016_S_1121
016_S_1138	016_S_1149	016_S_1263	016_S_1326	018_S_0043	018_S_0057	018_S_0080	018_S_0087
018_S_0103	018_S_0142	018_S_0155	018_S_0286	018_S_0335	018_S_0369	018_S_0406	018_S_0425
018_S_0450	018_S_0633	018_S_0682	020_S_0097	020_S_0213	020_S_0883	020_S_0899	020_S_1288
021_S_0141	021_S_0159	021_S_0178	021_S_0231	021_S_0273	021_S_0276	021_S_0332	021_S_0337
021_S_0343	021_S_0424	021_S_0626	021_S_0642	021_S_0647	021_S_0753	021_S_0984	021_S_1109
022_S_0004	022_S_0007	022_S_0014	022_S_0044	022_S_0066	022_S_0096	022_S_0129	022_S_0130
022_S_0219	022_S_0543	022_S_0544	022_S_0750	022_S_0924	022_S_0961	022_S_1097	022_S_1351
022_S_1366	022_S_1394	023_S_0031	023_S_0042	023_S_0058	023_S_0061	023_S_0078	023_S_0081
023_S_0083	023_S_0084	023_S_0093	023_S_0126	023_S_0139	023_S_0217	023_S_0331	023_S_0376
023_S_0388	023_S_0604	023_S_0613	023_S_0625	023_S_0855	023_S_0887	023_S_0916	023_S_0926
023_S_0963	023_S_1046	023_S_1104	023_S_1126	023_S_1190	023_S_1247	023_S_1262	023_S_1289
023_S_1306	024_S_0985	024_S_1063	024_S_1171	024_S_1307	024_S_1393	024_S_1400	027_S_0074
027_S_0116	027_S_0118	027_S_0120	027_S_0179	027_S_0256	027_S_0307	027_S_0403	027_S_0404
027_S_0408	027_S_0417	027_S_0461	027_S_0485	027_S_0644	027_S_0835	027_S_0850	027_S_1045
027_S_1081	027_S_1082	027_S_1213	027_S_1254	027_S_1277	027_S_1385	027_S_1387	029_S_0824
029_S_0836	029_S_0843	029_S_0845	029_S_0866	029_S_0871	029_S_0878	029_S_0914	029_S_0999
029_S_1038	029_S_1056	029_S_1073	029_S_1184	029_S_1218	029_S_1318	029_S_1384	031_S_0294
031_S_0321	031_S_0351	031_S_0554	031_S_0568	031_S_0618	031_S_0821	031_S_0830	031_S_0867
031_S_1066	031_S_1209	032_S_0095	032_S_0147	032_S_0187	032_S_0214	032_S_0400	032_S_0479
032_S_0677	032_S_0718	032_S_0978	032_S_1037	032_S_1101	032_S_1169	033_S_0511	033_S_0513
033_S_0514	033_S_0516	033_S_0567	033_S_0723	033_S_0724	033_S_0725	033_S_0733	033_S_0734
033_S_0739	033_S_0741	033_S_0889	033_S_0906	033_S_0920	033_S_0922	033_S_0923	033_S_1016
033_S_1086	033_S_1098	033_S_1116	033_S_1279	033_S_1281	033_S_1283	033_S_1284	033_S_1285
033_S_1308	033_S_1309	035_S_0033	035_S_0048	035_S_0156	035_S_0204	035_S_0292	035_S_0341
035_S_0555	035_S_0997	036_S_0576	036_S_0577	036_S_0656	036_S_0672	036_S_0673	036_S_0748
036_S_0759	036_S_0760	036_S_0813	036_S_0869	036_S_0945	036_S_0976	036_S_1001	036_S_1023
036_S_1135	036_S_1240	037_S_0150	037_S_0182	037_S_0303	037_S_0327	037_S_0377	037_S_0454
037_S_0467	037_S_0501	037_S_0539	037_S_0552	037_S_0566	037_S_0588	037_S_0627	037_S_1078
037_S_1225	037_S_1421	041_S_0125	041_S_0262	041_S_0282	041_S_0314	041_S_0407	041_S_0446
041_S_0549	041_S_0598	041_S_0679	041_S_0721	041_S_0898	041_S_1002	041_S_1010	041_S_1260
041_S_1368	041_S_1391	041_S_1411	041_S_1412	041_S_1418	041_S_1420	041_S_1423	041_S_1425
041_S_1435	051_S_1040	051_S_1072	051_S_1123	051_S_1131	051_S_1331	051_S_1338	052_S_0671
052_S_0951	052_S_0952	052_S_0989	052_S_1054	052_S_1168	052_S_1250	052_S_1251	052_S_1346
052_S_1352	053_S_0389	053_S_0507	053_S_0621	053_S_0919	053_S_1044	057_S_0464	057_S_0474

Supplementary Table S6. List of ADNI subjects' ID used in this study.

057_S_0643	057_S_0779	057_S_0818	057_S_0839	057_S_0934	057_S_0941	057_S_0957	057_S_1007
057_S_1217	057_S_1265	057_S_1269	057_S_1371	057_S_1373	057_S_1379	062_S_0535	062_S_0578
062_S_0690	062_S_0730	062_S_0768	062_S_0793	062_S_1099	062_S_1182	062_S_1294	062_S_1299
067_S_0019	067_S_0029	067_S_0038	067_S_0045	067_S_0056	067_S_0059	067_S_0076	067_S_0077
067_S_0098	067_S_0110	067_S_0176	067_S_0177	067_S_0243	067_S_0257	067_S_0284	067_S_0290
067_S_0336	067_S_0607	067_S_0812	067_S_0828	067_S_1185	067_S_1253	068_S_0109	068_S_0127
068_S_0210	068_S_0401	068_S_0442	068_S_0473	068_S_0476	068_S_0478	068_S_0802	068_S_0872
068_S_1075	068_S_1191	072_S_0315	072_S_1211	072_S_1380	073_S_0089	073_S_0311	073_S_0312
073_S_0386	073_S_0445	073_S_0518	073_S_0565	073_S_0746	073_S_0909	073_S_1357	082_S_0304
082_S_0363	082_S_0469	082_S_0640	082_S_0641	082_S_0761	082_S_0832	082_S_0928	082_S_1079
082_S_1119	082_S_1256	082_S_1377	094_S_0434	094_S_0489	094_S_0526	094_S_0531	094_S_0692
094_S_0711	094_S_0921	094_S_1015	094_S_1027	094_S_1090	094_S_1102	094_S_1164	094_S_1188
094_S_1241	094_S_1267	094_S_1293	094_S_1314	094_S_1330	094_S_1397	094_S_1398	094_S_1402
094_S_1417	098_S_0149	098_S_0160	098_S_0171	098_S_0172	098_S_0269	098_S_0288	098_S_0667
098_S_0884	098_S_0896	099_S_0040	099_S_0051	099_S_0054	099_S_0060	099_S_0090	099_S_0111
099_S_0291	099_S_0352	099_S_0372	099_S_0470	099_S_0492	099_S_0533	099_S_0534	099_S_0551
099_S_0880	099_S_0958	099_S_1034	099_S_1144	100_S_0006	100_S_0015	100_S_0035	100_S_0047
100_S_0069	100_S_0190	100_S_0296	100_S_0743	100_S_0747	100_S_0892	100_S_0930	100_S_0995
100_S_1062	100_S_1113	100_S_1154	100_S_1226	100_S_1286	109_S_0777	109_S_0876	109_S_0950
109_S_0967	109_S_1013	109_S_1014	109_S_1114	109_S_1157	109_S_1183	109_S_1192	109_S_1343
114_S_0166	114_S_0173	114_S_0228	114_S_0374	114_S_0378	114_S_0410	114_S_0416	114_S_0458
114_S_0601	114_S_0979	114_S_1103	114_S_1106	114_S_1118	116_S_0360	116_S_0361	116_S_0370
116_S_0382	116_S_0392	116_S_0487	116_S_0648	116_S_0649	116_S_0657	116_S_0752	116_S_0834
116_S_0890	116_S_1083	116_S_1232	116_S_1243	116_S_1249	116_S_1271	116_S_1315	121_S_1322
121_S_1350	123_S_0050	123_S_0072	123_S_0088	123_S_0091	123_S_0094	123_S_0106	123_S_0108
123_S_0113	123_S_0162	123_S_0298	123_S_0390	123_S_1300	126_S_0405	126_S_0506	126_S_0605
126_S_0606	126_S_0680	126_S_0708	126_S_0709	126_S_0784	126_S_0865	126_S_0891	126_S_1077
126_S_1187	126_S_1221	126_S_1340	127_S_0112	127_S_0259	127_S_0260	127_S_0393	127_S_0394
127_S_0397	127_S_0431	127_S_0622	127_S_0684	127_S_0754	127_S_0844	127_S_0925	127_S_1032
127_S_1140	127_S_1210	127_S_1382	127_S_1419	127_S_1427	128_S_0135	128_S_0138	128_S_0167
128_S_0188	128_S_0200	128_S_0205	128_S_0216	128_S_0225	128_S_0227	128_S_0229	128_S_0230
128_S_0245	128_S_0258	128_S_0266	128_S_0272	128_S_0310	128_S_0500	128_S_0517	128_S_0522
128_S_0528	128_S_0545	128_S_0608	128_S_0611	128_S_0715	128_S_0740	128_S_0770	128_S_0863
128_S_0947	128_S_1043	128_S_1088	128_S_1148	128_S_1242	128_S_1406	128_S_1407	128_S_1408
128_S_1409	128_S_1430	129_S_0778	129_S_1204	129_S_1246	130_S_0102	130_S_0232	130_S_0285
130_S_0289	130_S_0423	130_S_0449	130_S_0505	130_S_0783	130_S_0886	130_S_0956	130_S_0969
130_S_1200	130_S_1201	130_S_1290	130_S_1337	131_S_0123	131_S_0319	131_S_0384	131_S_0409
131_S_0436	131_S_0441	131_S_0457	131_S_0497	131_S_0691	131_S_1301	131_S_1389	132_S_0339
132_S_0987	133_S_0433	133_S_0488	133_S_0493	133_S_0525	133_S_0629	133_S_0638	133_S_0727
133_S_0771	133_S_0792	133_S_0912	133_S_0913	133_S_1031	133_S_1055	133_S_1170	136_S_0086
136_S_0107	136_S_0184	136_S_0186	136_S_0194	136_S_0195	136_S_0196	136_S_0299	136_S_0300
136_S_0426	136_S_0429	136_S_0579	136_S_0695	136_S_0873	136_S_0874	136_S_1227	137_S_0158
137_S_0283	137_S_0301	137_S_0366	137_S_0438	137_S_0443	137_S_0459	137_S_0481	137_S_0631
137_S_0668	137_S_0669	137_S_0686	137_S_0722	137_S_0796	137_S_0800	137_S_0825	137_S_0841
137_S_0972	137_S_0973	137_S_0994	137_S_1041	137_S_1414	141_S_0696	141_S_0697	141_S_0717
141_S_0726	141_S_0767	141_S_0790	141_S_0810	141_S_0851	141_S_0852	141_S_0853	141_S_0915
141_S_0982	141_S_1004	141_S_1024	141_S_1051	141_S_1052	141_S_1094	141_S_1137	141_S_1152
141_S_1231	141_S_1244	141_S_1245	141_S_1255	141_S_1378	941_S_1194	941_S_1195	941_S_1197
941_S_1202	941_S_1203	941_S_1295	941_S_1311	941_S_1363	002_S_2010	002_S_2073	005_S_2390
007_S_2058	007_S_2106	007_S_2394	009_S_2208	009_S_2381	011_S_2274	013_S_2324	013_S_2389
014_S_2185	014_S_2308	016_S_2007	016_S_2031	018_S_0055	018_S_2133	018_S_2138	018_S_2155
018_S_2180	021_S_2077	021_S_2100	021_S_2124	021_S_2125	021_S_2142	021_S_2150	022_S_2087
022_S_2167	022_S_2263	022_S_2379	023_S_2068	024_S_2239	027_S_2183	027_S_2219	027_S_2245

Supplementary Table S7. List of ADNI subjects' ID used in this study (continuation).

027_S_2336	029_S_2376	029_S_2395	031_S_2018	031_S_2022	031_S_2233	032_S_2119	032_S_2240
032_S_2247	035_S_2061	035_S_2074	036_S_2378	036_S_2380	052_S_2249	053_S_2357	053_S_2396
057_S_2398	067_S_2195	067_S_2196	067_S_2301	067_S_2304	068_S_2168	068_S_2171	068_S_2184
068_S_2187	068_S_2193	068_S_2194	068_S_2248	068_S_2315	068_S_2316	072_S_2026	072_S_2027
072_S_2037	072_S_2072	072_S_2083	072_S_2093	072_S_2116	072_S_2164	073_S_2153	073_S_2182
073_S_2190	073_S_2191	073_S_2225	073_S_2264	082_S_2099	082_S_2121	082_S_2307	094_S_2201
094_S_2216	094_S_2238	094_S_2367	098_S_2047	098_S_2052	098_S_2079	099_S_2042	099_S_2063
099_S_2146	099_S_2205	114_S_2392	123_S_2055	123_S_2363	126_S_2360	126_S_2405	126_S_2407
127_S_2213	127_S_2234	128_S_2002	128_S_2036	128_S_2045	128_S_2123	129_S_2332	129_S_2347
130_S_2373	130_S_2403	141_S_2210	141_S_2333	153_S_2109	153_S_2148	002_S_2043	003_S_2374
016_S_2284	109_S_2200	128_S_2130	128_S_2151	128_S_2220	941_S_2060	035_S_2199	072_S_2070
109_S_2237	109_S_2278	128_S_2003	128_S_2011	128_S_2057	128_S_2314	002_S_4171	002_S_4213
002_S_4219	002_S_4225	002_S_4229	002_S_4237	002_S_4251	002_S_4262	002_S_4264	002_S_4270
002_S_4447	002_S_4473	002_S_4521	002_S_4654	002_S_4746	002_S_4799	002_S_5018	002_S_5178
002_S_5230	002_S_5256	003_S_4081	003_S_4119	003_S_4152	003_S_4288	003_S_4350	003_S_4354
003_S_4373	003_S_4441	003_S_4524	003_S_4555	003_S_4644	003_S_4872	003_S_4892	003_S_4900
003_S_5130	003_S_5150	003_S_5154	003_S_5165	003_S_5187	003_S_5209	005_S_4168	005_S_4185
005_S_4707	005_S_4910	005_S_5038	005_S_5119	006_S_4150	006_S_4153	006_S_4192	006_S_4346
006_S_4357	006_S_4363	006_S_4449	006_S_4485	006_S_4515	006_S_4546	006_S_4679	006_S_4713
006_S_4867	006_S_4960	006_S_5153	007_S_4272	007_S_4387	007_S_4467	007_S_4488	007_S_4516
007_S_4568	007_S_4611	007_S_4620	007_S_4637	007_S_5196	007_S_5265	009_S_4324	009_S_4337
009_S_4359	009_S_4388	009_S_4530	009_S_4543	009_S_4564	009_S_4612	009_S_4741	009_S_4814
009_S_4903	009_S_4958	009_S_5000	009_S_5027	009_S_5037	009_S_5125	009_S_5147	009_S_5176
009_S_5224	009_S_5252	010_S_4345	010_S_4442	011_S_4075	011_S_4105	011_S_4120	011_S_4222
011_S_4235	011_S_4278	011_S_4366	011_S_4547	011_S_4827	011_S_4845	011_S_4893	011_S_4906
011_S_4912	011_S_4949	012_S_4012	012_S_4026	012_S_4094	012_S_4188	012_S_4643	012_S_5121
012_S_5157	013_S_4268	013_S_4395	013_S_4579	013_S_4580	013_S_4595	013_S_4616	013_S_4791
013_S_4917	013_S_4985	013_S_5071	013_S_5137	013_S_5171	014_S_4039	014_S_4058	014_S_4079
014_S_4080	014_S_4093	014_S_4263	014_S_4328	014_S_4401	014_S_4576	014_S_4577	014_S_4615
014_S_4668	016_S_4009	016_S_4097	016_S_4121	016_S_4353	016_S_4575	016_S_4583	016_S_4584
016_S_4591	016_S_4601	016_S_4638	016_S_4646	016_S_4688	016_S_4887	016_S_4902	016_S_4951
016_S_4952	016_S_4963	016_S_5007	016_S_5031	016_S_5032	016_S_5057	018_S_4257	018_S_4313
018_S_4349	018_S_4399	018_S_4400	018_S_4597	018_S_4696	018_S_4733	018_S_4809	018_S_4868
018_S_4889	018_S_5240	018_S_5250	018_S_5262	019_S_4252	019_S_4285	019_S_4293	019_S_4367
019_S_4477	019_S_4548	019_S_4549	019_S_4680	019_S_4835	019_S_5012	019_S_5019	019_S_5242
020_S_4920	020_S_5140	020_S_5203	021_S_4245	021_S_4254	021_S_4276	021_S_4335	021_S_4402
021_S_4419	021_S_4421	021_S_4558	021_S_4633	021_S_4659	021_S_4718	021_S_4744	021_S_4857
021_S_4924	021_S_5099	021_S_5129	021_S_5177	021_S_5194	021_S_5236	021_S_5237	022_S_4173
022_S_4266	022_S_4291	022_S_4320	022_S_4444	022_S_4805	022_S_4922	022_S_5004	023_S_4020
023_S_4034	023_S_4035	023_S_4115	023_S_4122	023_S_4164	023_S_4241	023_S_4243	023_S_4448
023_S_4501	023_S_4502	023_S_4796	023_S_5241	024_S_4084	024_S_4158	024_S_4169	024_S_4186
024_S_4223	024_S_4280	024_S_4392	024_S_4622	024_S_4674	024_S_4905	024_S_5054	024_S_5290
027_S_4729	027_S_4757	027_S_4801	027_S_4802	027_S_4804	027_S_4869	027_S_4873	027_S_4919
027_S_4926	027_S_4936	027_S_4938	027_S_4943	027_S_4955	027_S_4962	027_S_4964	027_S_4966
027_S_5079	027_S_5083	027_S_5093	027_S_5109	027_S_5110	027_S_5118	027_S_5127	027_S_5169
027_S_5170	027_S_5277	027_S_5288	029_S_4279	029_S_4307	029_S_4327	029_S_4384	029_S_4385
029_S_4585	029_S_4652	029_S_5135	029_S_5158	029_S_5166	029_S_5219	031_S_4005	031_S_4021
031_S_4024	031_S_4029	031_S_4032	031_S_4042	031_S_4149	031_S_4194	031_S_4203	031_S_4218
031_S_4474	031_S_4476	031_S_4496	031_S_4590	031_S_4947	032_S_4277	032_S_4348	032_S_4386
032_S_4429	032_S_4755	032_S_4823	032_S_4921	032_S_5263	032_S_5289	033_S_4176	033_S_4177
033_S_4179	033_S_4505	033_S_4508	033_S_5013	033_S_5017	033_S_5087	033_S_5198	033_S_5235
033_S_5259	035_S_4082	035_S_4085	035_S_4114	035_S_4256	035_S_4414	035_S_4464	035_S_4582
035_S_4783	035_S_4784	035_S_4785	036_S_4389	036_S_4430	036_S_4491	036_S_4538	036_S_4562

Supplementary Table S8. List of ADNI subjects' ID used in this study (continuation).

036_S_4714	036_S_4715	036_S_4736	036_S_4740	036_S_4820	036_S_4878	036_S_4894	036_S_4899
036_S_5063	036_S_5112	036_S_5210	036_S_5248	036_S_5271	036_S_5283	037_S_4001	037_S_4015
037_S_4028	037_S_4030	037_S_4071	037_S_4146	037_S_4214	037_S_4302	037_S_4308	037_S_4381
037_S_4410	037_S_4432	037_S_4706	037_S_4750	037_S_4770	037_S_4879	037_S_5126	037_S_5162
037_S_5222	041_S_4004	041_S_4014	041_S_4037	041_S_4041	041_S_4051	041_S_4060	041_S_4138
041_S_4143	041_S_4200	041_S_4271	041_S_4427	041_S_4510	041_S_4513	041_S_4629	041_S_4720
041_S_4874	041_S_4876	041_S_4877	041_S_4974	041_S_4989	041_S_5026	041_S_5078	041_S_5082
041_S_5097	041_S_5100	041_S_5131	041_S_5141	041_S_5204	041_S_5244	041_S_5253	051_S_4929
051_S_4980	051_S_5285	051_S_5294	052_S_4626	052_S_4807	052_S_4885	052_S_4944	052_S_4945
052_S_4959	052_S_5062	053_S_4557	053_S_4578	053_S_4661	053_S_4813	053_S_5070	053_S_5202
053_S_5208	053_S_5272	053_S_5287	053_S_5296	057_S_4888	057_S_4897	057_S_4909	057_S_5199
057_S_5292	067_S_4054	067_S_4072	067_S_4184	067_S_4212	067_S_4310	067_S_4728	067_S_4767
067_S_4782	067_S_4918	067_S_5159	067_S_5160	067_S_5205	068_S_4061	068_S_4067	068_S_4134
068_S_4174	068_S_4217	068_S_4274	068_S_4332	068_S_4340	068_S_4424	068_S_4431	068_S_4859
068_S_4914	068_S_4968	068_S_5146	068_S_5206	070_S_4692	070_S_4708	070_S_4719	070_S_4793
070_S_4856	070_S_5040	072_S_4007	072_S_4057	072_S_4063	072_S_4102	072_S_4103	072_S_4131
072_S_4206	072_S_4226	072_S_4383	072_S_4390	072_S_4391	072_S_4394	072_S_4445	072_S_4462
072_S_4465	072_S_4522	072_S_4539	072_S_4610	072_S_4613	072_S_4694	072_S_4769	072_S_4871
072_S_4941	072_S_5207	073_S_4155	073_S_4216	073_S_4259	073_S_4300	073_S_4311	073_S_4312
073_S_4360	073_S_4382	073_S_4393	073_S_4403	073_S_4443	073_S_4540	073_S_4552	073_S_4559
073_S_4614	073_S_4739	073_S_4762	073_S_4795	073_S_4825	073_S_4853	073_S_4986	073_S_5016
073_S_5023	073_S_5090	073_S_5167	073_S_5227	082_S_4090	082_S_4208	082_S_4224	082_S_4244
082_S_4339	082_S_4428	082_S_5014	082_S_5029	082_S_5184	082_S_5278	082_S_5279	082_S_5282
094_S_4089	094_S_4162	094_S_4234	094_S_4282	094_S_4434	094_S_4459	094_S_4503	094_S_4560
094_S_4630	094_S_4649	094_S_4737	094_S_4858	098_S_4003	098_S_4018	098_S_4050	098_S_4059
098_S_4201	098_S_4215	098_S_4275	098_S_4506	099_S_4022	099_S_4076	099_S_4086	099_S_4104
099_S_4157	099_S_4202	099_S_4205	099_S_4463	099_S_4475	099_S_4480	099_S_4498	099_S_4565
099_S_4994	100_S_4469	100_S_4512	100_S_4556	100_S_5075	100_S_5091	100_S_5096	100_S_5102
100_S_5106	100_S_5280	109_S_4260	109_S_4380	109_S_4455	109_S_4499	109_S_4531	109_S_4594
114_S_4379	114_S_4404	114_S_5047	114_S_5234	116_S_4010	116_S_4043	116_S_4092	116_S_4167
116_S_4175	116_S_4195	116_S_4199	116_S_4209	116_S_4338	116_S_4453	116_S_4483	116_S_4625
116_S_4635	116_S_4855	116_S_4898	123_S_4096	123_S_4127	123_S_4170	123_S_4362	123_S_4526
123_S_4780	123_S_4806	123_S_4904	126_S_4458	126_S_4494	126_S_4507	126_S_4514	126_S_4675
126_S_4686	126_S_4712	126_S_4743	126_S_4891	126_S_4896	126_S_5214	126_S_5243	127_S_4148
127_S_4197	127_S_4198	127_S_4210	127_S_4240	127_S_4301	127_S_4500	127_S_4604	127_S_4624
127_S_4645	127_S_4765	127_S_4843	127_S_4844	127_S_4928	127_S_4940	127_S_4992	127_S_5028
127_S_5056	127_S_5058	127_S_5067	127_S_5095	127_S_5132	127_S_5185	127_S_5200	127_S_5218
127_S_5228	127_S_5266	128_S_4553	128_S_4571	128_S_4586	128_S_4599	128_S_4603	128_S_4607
128_S_4609	128_S_4636	128_S_4653	128_S_4671	128_S_4742	128_S_4745	128_S_4772	128_S_4774
128_S_4792	128_S_4832	128_S_4842	128_S_5066	128_S_5123	129_S_4073	129_S_4220	129_S_4287
129_S_4369	129_S_4371	129_S_4396	129_S_4422	130_S_4250	130_S_4294	130_S_4343	130_S_4352
130_S_4405	130_S_4415	130_S_4417	130_S_4468	130_S_4542	130_S_4589	130_S_4605	130_S_4641
130_S_4660	130_S_4730	130_S_4817	130_S_4883	130_S_4925	130_S_4971	130_S_4982	130_S_4984
130_S_4990	130_S_4997	130_S_5006	130_S_5059	130_S_5142	130_S_5175	130_S_5258	131_S_5138
131_S_5148	135_S_4281	135_S_4309	135_S_4356	135_S_4406	135_S_4446	135_S_4489	135_S_4566
135_S_4598	135_S_4657	135_S_4676	135_S_4689	135_S_4722	135_S_4723	135_S_4863	135_S_4954
135_S_5015	135_S_5113	135_S_5269	135_S_5273	135_S_5275	136_S_4189	136_S_4269	136_S_4408
136_S_4433	136_S_4517	136_S_4993	137_S_4211	137_S_4258	137_S_4299	137_S_4303	137_S_4331
137_S_4351	137_S_4466	137_S_4482	137_S_4520	137_S_4536	137_S_4587	137_S_4596	137_S_4623
137_S_4631	137_S_4632	137_S_4672	137_S_4678	137_S_4756	137_S_4815	137_S_4816	137_S_4852
137_S_4862	141_S_4053	141_S_4160	141_S_4232	141_S_4423	141_S_4426	141_S_4438	141_S_4456
141_S_4711	141_S_4803	141_S_4907	141_S_4976	153_S_4077	153_S_4125	153_S_4133	153_S_4139
153_S_4151	153_S_4159	153_S_4172	153_S_4297	153_S_4372	153_S_4621	153_S_4838	153_S_5261

Supplementary Table S9. List of ADNI subjects' ID used in this study (continuation).

153_S_5267	941_S_4036	941_S_4066	941_S_4100	941_S_4187	941_S_4255	941_S_4292	941_S_4365
941_S_4376	941_S_4377	941_S_4420	941_S_4764	941_S_5193	070_S_4798	022_S_4196	035_S_6739
037_S_6115	037_S_6125	037_S_6222	005_S_6084	007_S_6120	002_S_6103	019_S_6635	019_S_6668
011_S_6465	109_S_6220	099_S_6097	067_S_6117	114_S_6113	130_S_6027	130_S_6105	130_S_6111
135_S_6110	123_S_6118	127_S_6024	341_S_6605	141_S_6116	168_S_6131	168_S_6413	941_S_6471

Supplementary Table S10. List of ADNI subjects' ID used in this study (continuation).

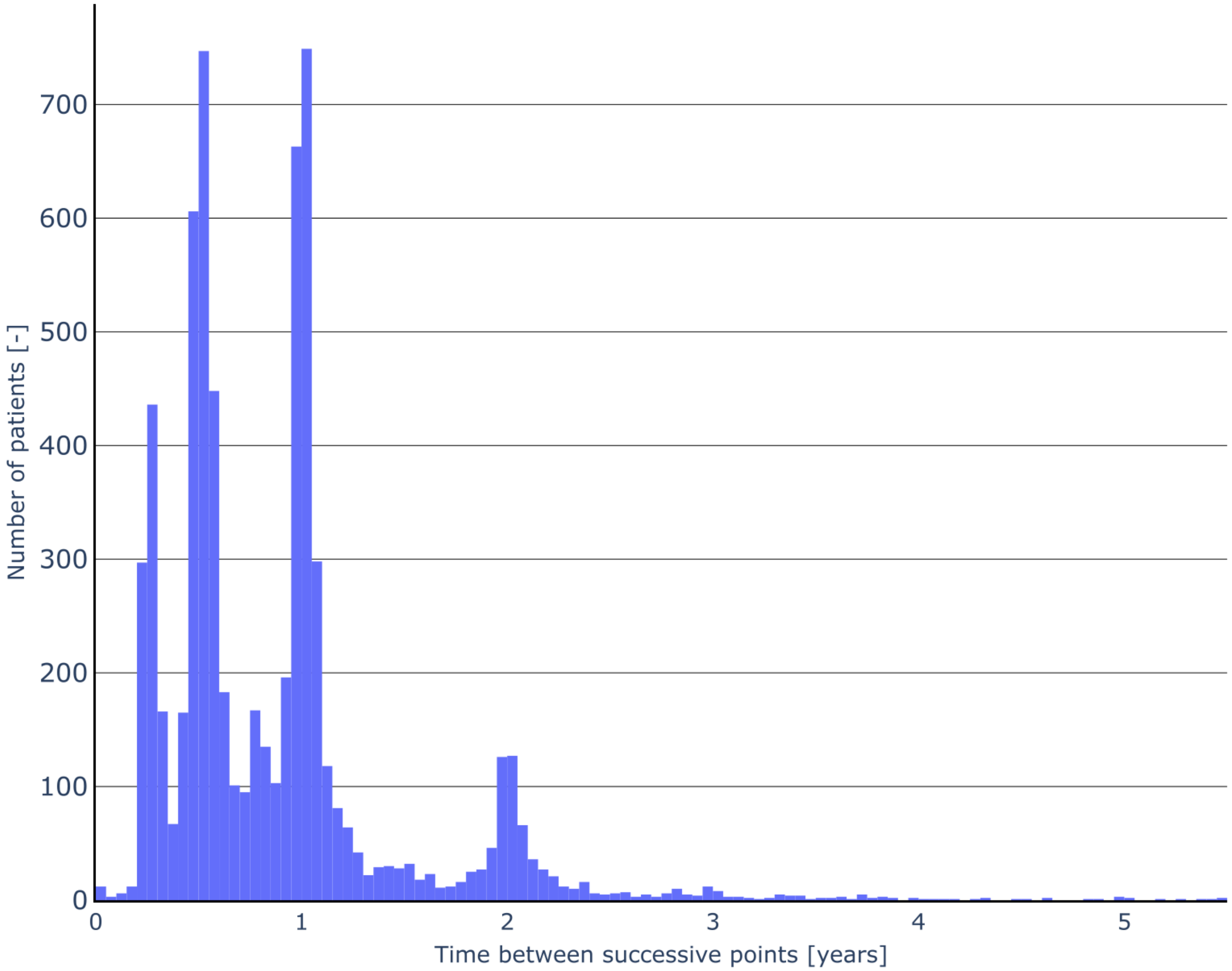

Supplementary Figure S1. Time step distribution for the ADNI database. The distribution of time between successive subjects' scan, for ADNI database.

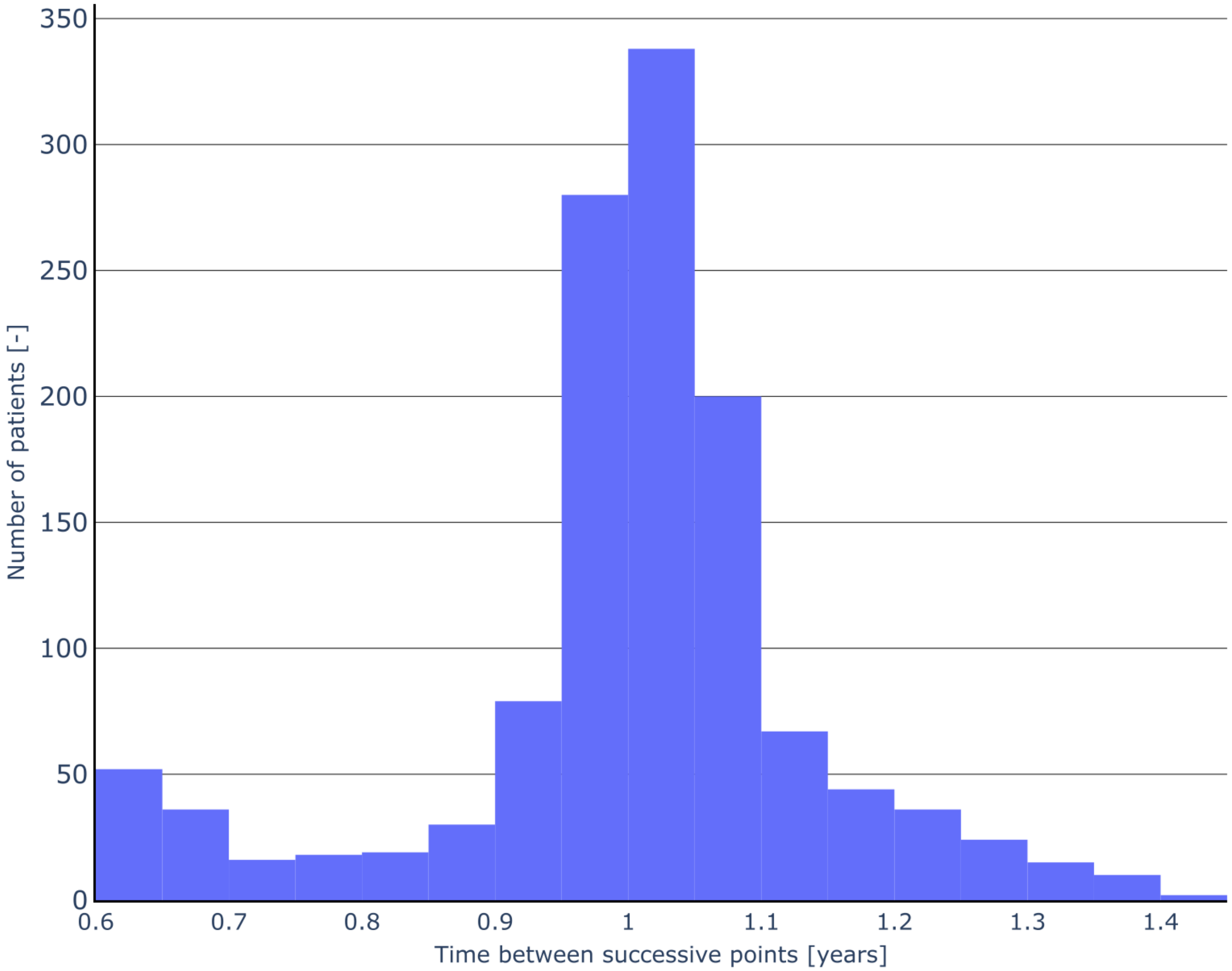

Supplementary Figure S2. Time step distribution for the standardized ADNI database. The distribution of time between successive subjects' scan, for ADNI database after having standardized this Δt. The standardization consist of removing data points so that the time between scan is always 1 ± 0.4 year. Subjects with one time point are also removed.

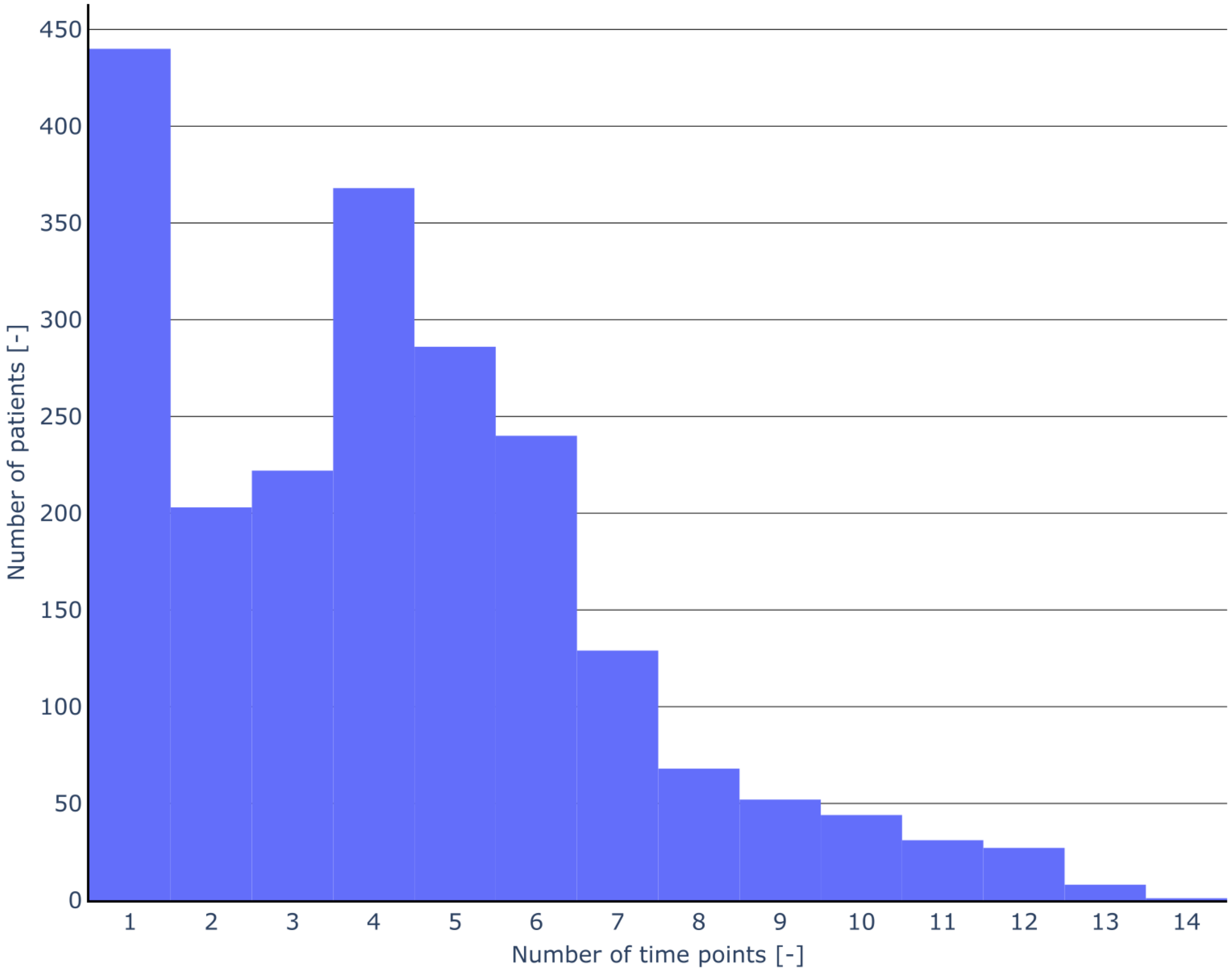

Supplementary Figure S3. The distribution of the number of scans in ADNI.

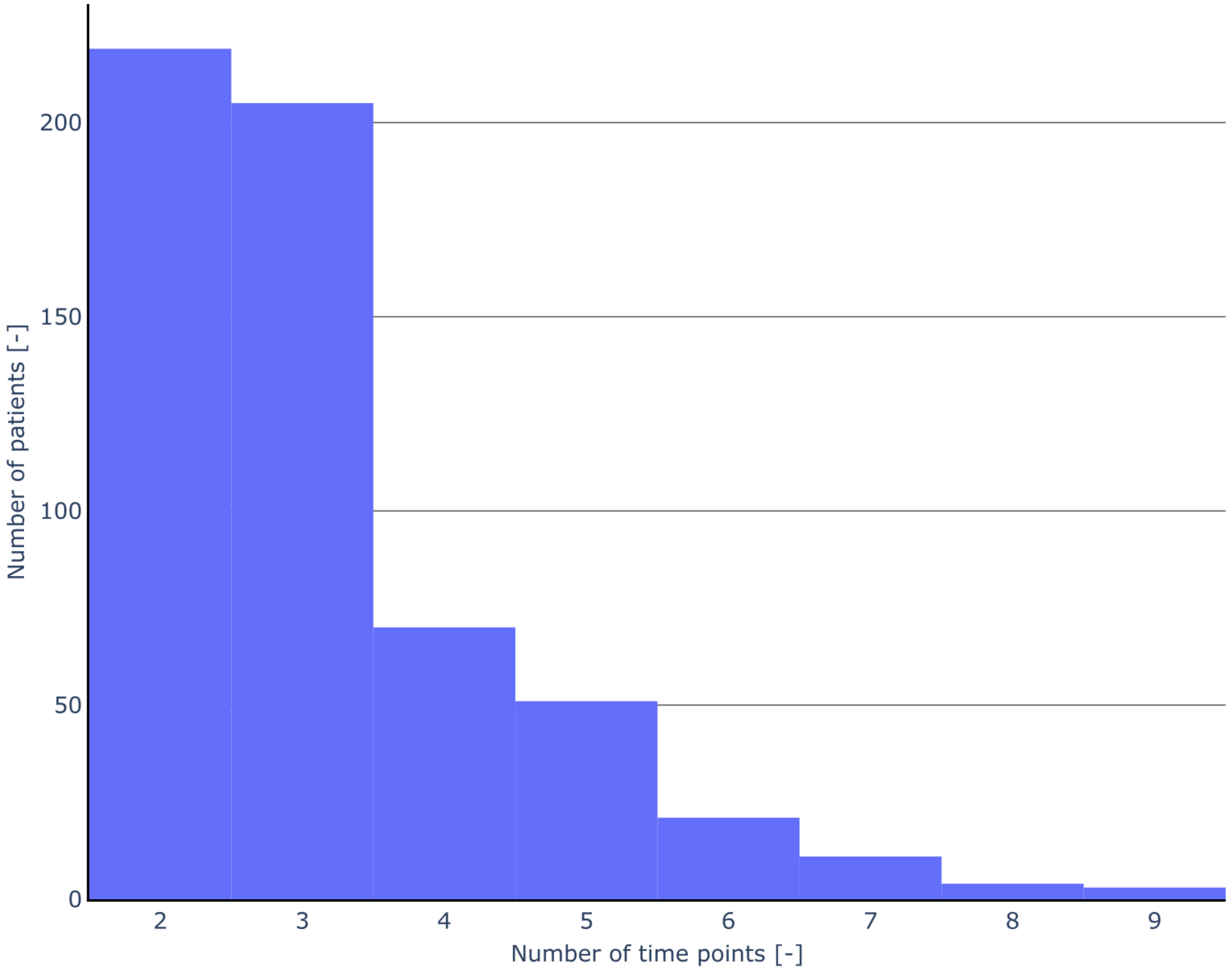

Supplementary Figure S4. The distribution of the number of scans in ADNI after standardization. Refer to Supplementary Figure S2 for a description of the standardization

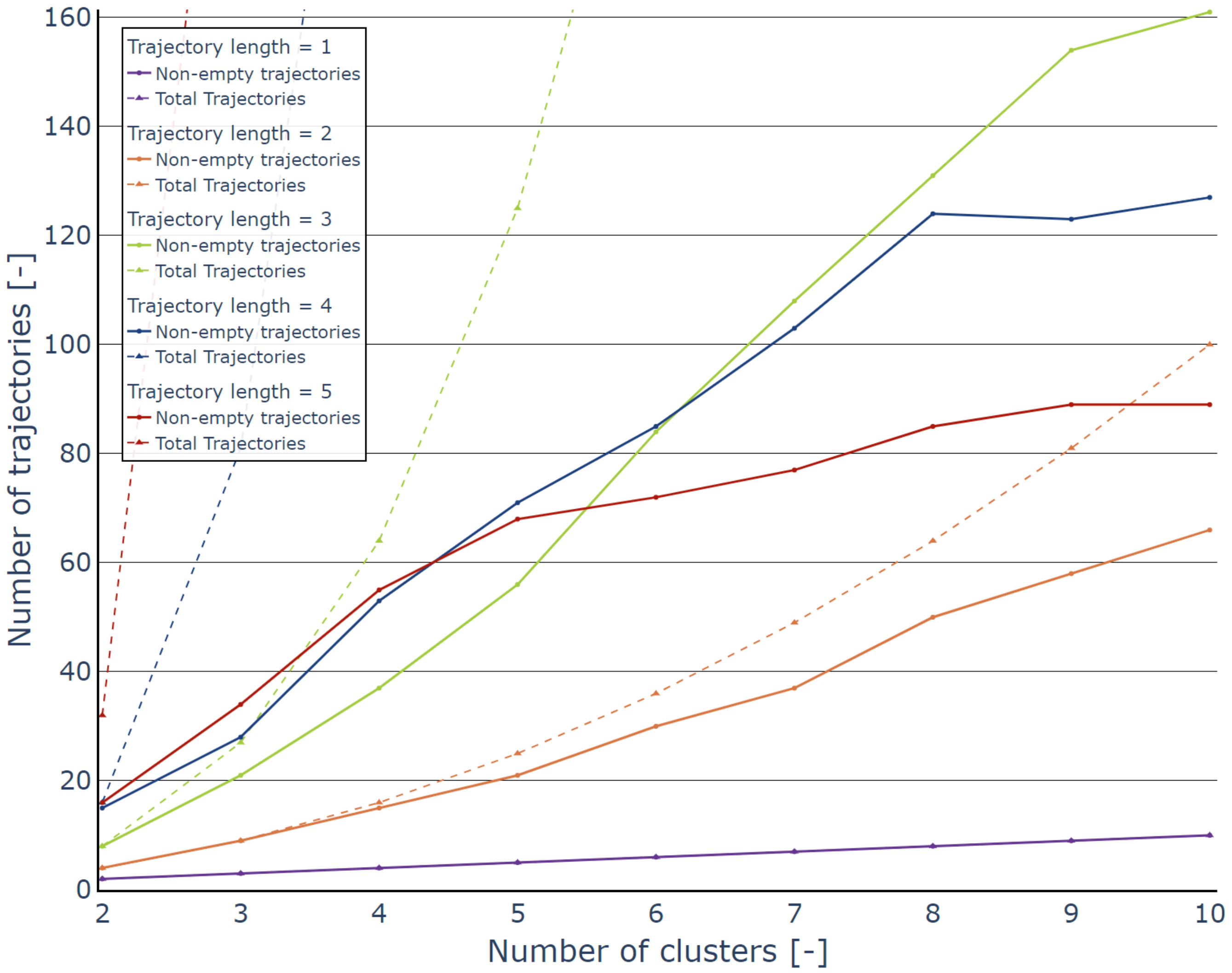

Supplementary Figure S5. The population in each trajectory by number of clusters (C). These values were obtained from the embedding with `n_neighbors`=20 and `min_dist`=0. The trajectory lengths (l) are colorcoded. The full lines are the number of trajectories that host at least one subject, by C, while the dashed lines are the total number of trajectories available as a function of C, also given by C^l. It is possible to see that from a trajectory length of 4, increasing the value reduces the number of trajectories that can be use. For a length of 4, the number of trajectories falls below the length of 3 after 6 clusters and the same happens for the curve with trajectory length of 5 after 5 clusters.

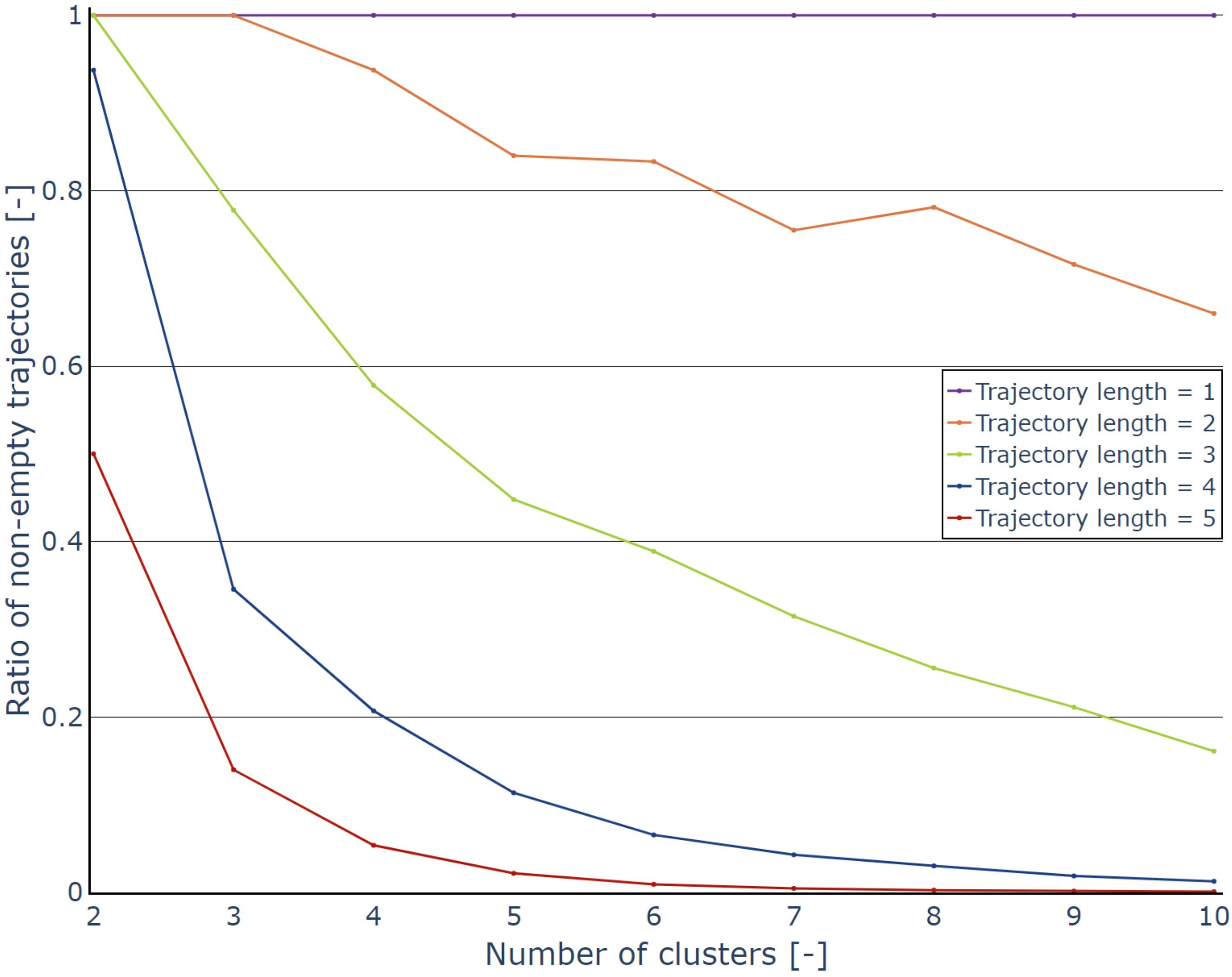

Supplementary Figure S6. The ratio of trajectories with at least one subject as a function of the number of clusters (C). These values were obtained from the embedding with `n_neighbors`=20 and `min_dist`=0. The trajectory lengths (l) are colorcoded. Similarly to Supplementary Figure S5, this figure showcases that too high l and C has diminishing returns. For $l \geq 4$ the ratio falls quickly under 20% and reaches $\approx$ 1% soon after. With the ADNI data, a maximum l of 3 seems to minimise the number of unpopulated trajectories
